# USP13 dictates Ran turnover and vulnerability to ferroptosis in diffuse large B cell lymphoma (DLBCL)

**DOI:** 10.1038/s41419-025-08207-6

**Published:** 2025-11-28

**Authors:** Xuan Qiao, Xingmeng Yang, Yuanhao Diao, Qiuxiang Li, Xianhuo Wang, Chong Li, Zailin Yang, Wee Joo Chng, Boheng Li

**Affiliations:** 1 College of Pharmaceutical Sciences, Southwest University, Chongqing, China; 2 Tianjin Medical University Cancer Institute and Hospital, Tianjin, China; 3 Tianjin’s Clinical Research Center for Cancers, Tianjin, China; 4 The Sino-US Center for Lymphoma and Leukemia Research, Tianjin, China; 5 Department of Hematology-Oncology, Chongqing University Cancer Hospital, Chongqing, China; 6 Department of Medicine, Yong Loo Lin School of Medicine, National University of Singapore, Singapore, Singapore; 7 Cancer Science Institute of Singapore, Yong Loo Lin School of Medicine, National University of Singapore, Singapore, Singapore; 8 Department of Hematology-Oncology, National University Cancer Institute of Singapore, National University Health System, Singapore, Singapore

**Keywords:** B-cell lymphoma, Ubiquitylation, Cell death

## Abstract

Diffuse large B-cell lymphoma (DLBCL) is one of the most common and lethal B-cell malignancies worldwide, with high relapse rate after standard treatment, and the relapsed cases are often difficult to treat. Ubiquitination has a pivotal role in cellular protein homeostasis and tumorigenic deubiquitinases (DUBs) stabilize oncoproteins during carcinogenesis. In this study, we have identified USP13 as one of the abnormally-overexpressed and stage-related DUBs in DLBCL critical to disease pathogenesis. Employing LC-MS/MS, Ran GTPase was characterized as potential substrate of USP13, with the interaction between Ran and USP13 modeled by Alphafold3. USP13 co-overexpressed, co-localized and correlated with Ran at protein level in DLBCL patient samples and cell lines. Pharmacological inhibition and genetic manipulation of USP13 modulated Ran protein stability and K11-linked ubiquitination level of Ran. By RNA-seq, pharmacological inhibition of USP13 via Spautin-1 treatment in DLBCL revealed downstream targets in NF-κB and Notch pathways. Suggested by the DRESIS Database, NF-κB and Notch signaling cascades are key to the resistance of Doxorubicin (Dox) and cyclophosphamide (CTX) within the standard R-CHOP regimen of DLBCL hinting benefits of combining Spautin-1 with Dox or CTX. Further in-vitro and in-vivo experiments confirmed Spautin-1 synergized with Dox or CTX to initiate ferroptosis in DLBCL with few toxicities to main organs by mediating ROS, GSH, MDA and ultimately LPO. Altogether, these results demonstrate USP13 deubiquitinates Ran in DLBCL, and Spautin-1 well synergizes with Dox or CTX to initiate ferroptosis. Combinational treatment with Spautin-1 and Dox or CTX may represent an effective approach and therapeutic hope to combat with DLBCL.

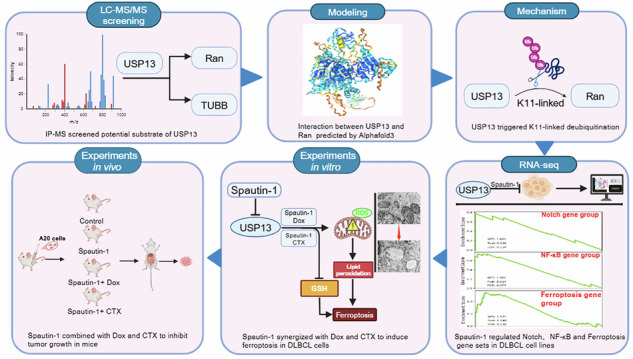

## Introduction

Diffuse large B cell lymphoma (DLBCL) represents one of the most common and aggressive lymphoid cancers in adults accounting for roughly 30% of non-Hodgkin’s lymphoma worldwide [1]. Pathological observations and genetic profiling have led to the characterization of DLBCL subtypes, namely germinal center B-cell DLBCL, activated B-cell DLBCL, and not otherwise specified DLBCL [2]. Genetic abnormalities leading to chronic NF-κB and Notch activations are enriched in this malignancy and are well-acknowledged as criminals of DLBCL proliferation and infiltration [3, 4]. Upon treatment with standard first-line therapeutics, one-third of DLBCL patients are refractory, and part of the remaining patients relapse over time [5]. New-generation therapies like proteasome inhibitors and PD-1 monoclonal antibody show more extensive effectiveness, but the success is still limited to specific genetic background of DLBCL patients [6–8], underpinning the need for further in-depth mechanistic and translational studies.

Protein ubiquitination is one of the sophistically orchestrated post-translational modification processes referring to the transfer of ubiquitin to the lysine or methionine residue of the substrate protein. Ubiquitination mostly alters protein stability and sequentially requires E1 activating enzyme, E2 conjugating enzyme, and E3 ligase to relay the ubiquitin [9]. Deubiquitinases (DUBs) reverse the ubiquitination process, resulting in protein stability recovery. Overexpression of the DUB USP13 has been identified in gastric [10], kidney [11], lung [12, 13], ovarian [13], breast [14] and cervical [15] cancers functioning as oncoprotein or tumor suppressor depending on specific cancerous context, and regulates the turnover of several key players in cancers including MCL-1 [13, 15], MYC [12], FASN [16], ZHX2 [11] and PTEN [14]. In contrast, the role of USP13 in DLBCL has merely been defined.

Ferroptosis is a mode of metal-dependent cell death triggered by the toxic accumulation of iron-dependent lipid peroxidation and high load of oxidative stress. Ferroptosis is characterized by mitochondria shrinkage and mitochondrial cristae reduction, morphologically and mechanistically different from other forms of cell death like apoptosis and autophagy [17]. Numerous studies have suggested that chemo-resistant cancer cells, especially those with a high speed of proliferation and prone to metastasis and infiltration, are particularly vulnerable to ferroptosis [18]. Given the patient refraction and aggressiveness of DLBCL, pharmacological induction of ferroptosis may be a useful avenue for the treatment of this malignancy.

In this study, employing LC-MS/MS, we identified Ran GTPase (hereafter refer to as Ran) as the potential substrate of USP13 in DLBCL via affinity purification and proteome-wide study. Bioinformatic analysis showed overexpression and correlation of USP13 and Ran as well as their role in DLBCL progression. Further Alphafold3 prediction and wet-bench experiments confirmed Ran as a bona-fide substrate of USP13 and delineated how USP13-Ran cascade modulated DLBCL pathogenesis. The USP13 inhibitor Spautin-1 induced ferroptosis in DLBCL through the regulation of NF-κB and Notch signaling and well synergized with conventional DLBCL chemo-agents, validated by both in-vivo and in-vitro experiments.

## Materials and methods

### Cell culture

The DLBCL cell lines SU-DHL-2, Farage, TMD8, U2932, WSU-DLCL2, and A20 were maintained in RPMI 1640 medium (Gibco) supplemented with 10% fetal bovine serum (FBS, Gibco). The Human normal B lymphocytes HMy2.CIR and the DLBCL cell lines OCI-LY3, OCI-LY1, and OCI-LY7 were maintained in IMDM medium (Gibco) supplemented with 10% FBS (Gibco). HEK293T cells were cultured in Dulbecco’s modified Eagle medium (Gibco) supplemented with 10% FBS (Gibco). All these cells were incubated at 37 °C with 5% CO_2_. All cell lines have been authenticated by the vendor or by our own group.

### Antibodies and drugs

Primary antibodies and drugs used in this study include: USP13 (Santa Cruz sc-514416, 1:1000), Ran (Santa Cruz sc-271376, 1:1000), normal mouse IgG (Santa Cruz sc-2025), GAPDH (Santa Cruz sc-47724, 1:1000), Histone H3 (Cell Signaling Technology 4499 T, 1:2000), β-Tubulin (Cell Signaling Technology 2128S), GFP (Santa Cruz sc-9996, 1:1000), HA-Tag (Santa Cruz sc-7392, 1:1000), Ubiquitin (Santa Cruz sc-8017, 1:1000), COX-IV (Wanleibio WL02203, 1:1000), Spautin-1 (Targetmol, T1937), MG132 (Targetmol, T2154), Cyclophosphamide hydrate (Targetmol, T0707), Doxorubicin hydrochloride (Targetmol, T1020) and erastin (Targetmol, T1765). Cycloheximide was obtained from the Chongqing Cancer Hospital.

### Constructs and siRNA transfection

For DLBCL cell lines, transfections with constructs and small interfering RNAs (siRNAs) were performed using the Neon Transfection System (Invitrogen) according to the manufacturer’s instructions. For HEK293T cells, the LIPOFECTAMINE3000 (Invitrogen) was used for constructs transfections. The pLenti-CMV-Flag-USP13-GFP-Puro, pLenti-CMV-Flag-USP13-C345A-GFP-Puro, pcDNA3.1( + )-HA-Ran, and pcDNA empty vector were purchased from Public Protein/Plasmid Library. The USP13 siRNA was acquired from Qingke. The Prk5-HA-Ub, Prk5-HA-K11-Ub, Prk5-HA-K48-Ub, and Prk5-HA-K63-Ub plasmids were kind gifts from Prof Hong Zhu (Zhejiang University, China). The sequences of siRNAs are listed in Table 1.

**Table 1:** siRNA details.

Gene	sequence
USP13	CAUGUGCGAGAGAAGGUAA (#1)
	GCUUAUGAACUAACGAGAA (#3)

### ShRNAs and lentivirus production

Lentiviral clones expressing USP13 shRNA (shUSP13) were purchased from Qingke. Viral particles were produced in HEK293T cells with the helper plasmids (Qingke). In general, plasmids psPAX2, pMD2.G, and shRNA constructs were co-transfected into HEK293T cells. Then the cells were incubated at 37 °C, and the medium was replaced after 24 h. And the virus-containing supernatant was collected 48 h after transfection. The viral stocks were concentrated by precipitation with Millipore Amicon Ultra (Merck). For virus transduction, the cells were mixed with virus-containing supernatants supplemented with polybrene (Biotopped). In addition, cells infected by virus-containing medium were selected later with puromycin.

### Cell viability assay

The cell survival was measured with the Cell Counting Kit-8 (Beyotime) according to the manufacturer’s instructions.

### Immunoblot

DLBCL or HEK293T cells were lysed with RIPA buffer (Yamei) and then sonicated by the Ultrasonic Homogenizer (SCIENTZ, China), followed by centrifugation to obtain protein supernatant. The protein concentration was measured with BCA kit (Dingguo). Then the immunoblot was carried out as previously described [19].

### Co-immunoprecipitation (Co-IP)

Antibodies were added into the cell lysates, and the lysates were rotated at 4 °C overnight after centrifugation for clarification. The lysates were mixed with the protein A/G beads (Invitrogen 10004D), and incubated at 4 °C for 1 h. Then the beads were sequentially washed with lysis buffer and PBS, and SDS loading buffer (Baoguang) was added to the beads, which were then heated at 100 °C for 10 min before loading for immunoblots.

### Quantitative real-time PCR (qRT-PCR)

Total RNA was extracted using Trizol reagent (Beyotime), and was reverse-transcribed with the Fastking RT kit (Tiangen). qRT-PCR was performed using SYBR Green mix (Tiangen) on a ConnectTM Real-Time System (CFX, USA). The primers used for qRT-PCR are indicated in Table 2. Relative quantitation analysis of gene expression data was conducted according to the 2 − ΔΔCt method [20].

**Table 2:** Primer sequences.

Gene	Sequences	
USP13	Forward	TCTACAAGAACGAGTGCGCC
	Reverse	TTGGTAACGCTCCACCAGAC
Ran	Forward	GAGCCCCAGGTCCAGTTCAAA
	Reverse	ACACCCAAGGTGGCTACATAC
GAPDH	Forward	GCTGGCGCTGAGTAGTCGTGGAGT
	Reverse	CACAGTCTTCTGGGTGGCAGTGATGG
NQO1	Forward	TGAAAGGCTGGTTTGAGCGA
	Reverse	AGCACTGCCTTCTTACTCCG
ALOX12	Forward	GTGCTGGCAGGATGATGAGT
	Reverse	CTCTCCTCGGATCACGTTGG
Notch1	Forward	CAGAGGCGTGGCAGACTAT
	Reverse	CGGCACTTGTACTCCGTCAG
Hes1	Forward	GGCTAAGGTGTTTGGAGGCT
	Reverse	GGTGGGTTGGGGAGTTTAGG
BIRC3	Forward	AAGACTGGGCTTGTCCTTGC
	Reverse	TCCCGAGATTAGACTAAGTCCCTT
TNFAIP3	Forward	TCCTCAGGCTTTGTATTTGAGC
	Reverse	TGTGTATCGGTGCATGGTTTTA
TNFRSF14	Forward	CCAAGTGCAGTCCAGGTTAT
	Reverse	ATTGAGGTGGGCAATGTAGG
CD80	Forward	GGGAAATGTCGCCTCTCTGA
	Reverse	GCTCACGTAGAAGACCCTCC
CD44	Forward	TGACAACGCAGCAGAGTAATTC
	Reverse	TTCCACCTGTGACATCATTCCT

### Immunohistochemistry

For patient sample staining, 5 samples from pathologically diagnosed DLBCL patients were obtained from the Chongqing Cancer Hospital. The tissue slides were deparaffinized, treated with 3% H_2_O_2_ for 10 min, autoclaved in 10 mM citric sodium (pH 6.0) for 30 min to unmask antigens, rinsed in PBS, and then incubated with the primary antibodies against Ran (Santa Cruz, sc-271376, 1:250) or USP13 (Santa Cruz, sc-514416, 1:250) at 4 °C overnight, followed by incubation with biotinylated secondary antibody for 1 h at room temperature. Signal amplification and detection were performed using the DAB system according to the manufacturer’s instructions.

For animal cancerous tissue staining, dewaxed sections were obtained after tissue embedding. The slices were washed 3 times with PBS to repair the antigen. To block endogenous peroxidase, the slices were incubated in 3% hydrogen peroxide solution for 25 min away from light. The tissues were then covered with 3% BSA at room temperature for 30 min and incubated at 4 °C overnight with the primary antibody. After eluting the primary antibody with PBS, the secondary antibody of the corresponding species was incubated for 50 min. Then the secondary antibody was eluted with PBS, followed by color development by DAB and hematoxylin for 3 min. Finally, the images were viewed under a microscope, and the staining was quantified via ImageJ.

### Nuclear, cytoplasmic, and mitochondrial protein extraction

The Nuclear and Cytoplasmic Protein Extraction Kit (Beyotime) as well as the Cytoplasmic and Mitochondrial Protein Extraction Kit (Beyotime) were used for the extraction based on the manufacturer’s protocols, and the lysates were used for immunoblots.

### Liquid chromatography-mass spectrometry-mass spectrometry (LC–MS/MS) analysis

HEK293T cells in culture 48 h after transfection were subject to cell harvesting and lysis. And co-IP was performed using the lysate with USP13 pull-down until beads washing. Then 50 mM ammonium bicarbonate was added to the beads to a final volume of 100 μL. For proteome-wide analysis, the cell lysis was directly used for procedures afterwards. All these procedures were similar to our description before [19]. First, the samples were reduced by DTT at 56 °C for 1 h and alkylated by IAM at 25 °C in the dark for 40 min. Trypsin was added to the protein solution at a ratio of 1:100 for 4 h, and the mixtures were incubated at 37 °C for digestion overnight. After digestion, the peptide was desalted using a self-priming desalting column, and the solvent was evaporated in a vacuum centrifuge at 45 °C. Subsequently, the peptide was dissolved (0.1% formic acid, 2% acetonitrile), vortexed thoroughly, centrifuged at 13,200 rpm for 10 min at 4 °C, and the supernatant was transferred to the sample tube for mass spectrometry analysis. The LC–MS/MS analysis was performed on an Easy-nLC1200 System coupled with a Q Exactive™ Hybrid Quadrupole-Orbitrap™ Mass Spectrometer (Thermo Fisher, USA). 5 μL of each sample was loaded onto a C18 PepMap100 trap column and eluted on an Acclaim PepMap RPLC analytic column. A 66 min gradient procedure for each single analysis was performed as follows: 4–8% B for 2 min, 8–28% B for 43 min, 28–40% B for 10 min, 40–95% B for 1 min and 95–95% B for 10 min (A: 0.1% formic acid in water, B: 0.1% formic acid in water-acetonitrile (1:4)). The total flow rate was set at 600 nL/min. For MS analysis, MS resolution was set to 70,000 at 400 m/z, and the MS precursor m/z scan ranged from m/z 300 to 1800. MS/MS parameters were set as follows: production scan Range, m/z 50; activation type, HCD; normalized collision energy, 28.0; activation time, 66.00. Next, the raw MS files were analyzed and searched against the protein database based on the species of the samples using MaxQuant (1.6.2.10). The parameters were set as follows: the protein modifications were carbamidomethylation (C) (fixed), oxidation (M) (variable), Acetyl (Protein N-term) (variable); the enzyme specificity was set to trypsin; the maximum missed cleavages were set to 2; the precursor ion mass tolerance was set to 20 ppm, and MS/MS tolerance was 20 ppm. Only highly confident identified peptides were chosen for downstream protein identification analysis.

### Xenograft models

NOD-SCID (Jicuiyaokang) mice were maintained under pathogen-free conditions in the institutional SPF animal facility in accordance with the institutional guidelines. And A20 cells mixed with Matrigel (BD Biocoat) were injected subcutaneously (3 × 10^6^ cells per mouse, 4 mice per group) into 4- to 6-week-old female mice. Spautin-1, Doxorubicin (Dox), and Cyclophosphamide (CTX) were dissolved in 2% DMSO plus 40% PEG 300 and 5% Tween 80. Drug treatment began once the tumor volume reached 50–100 mm³. The mice were randomly and blindly allocated, and treated as vehicle, Spautin-1 (40 mg/kg, i.p., once every other day), Dox (2 mg/kg, i.g., once every three days), CTX (25 mg/kg, i.g., once every three days), alone or in combination. Tumor volume was determined by caliper measurements every 2 days and calculated using the formula (length × width squared)/2 [21].

### HE staining

After fixation and embedding, the tissues were dewaxed and frozen at -20 °C. Then the paraffin sections were fixed with tissue fixation solution for 15 min, and stained with hematoxylin solution for 3-5 min and dyed with eosin for 15 s. Slices were dewaxed again and sealed with neutral gum. Finally, the images were observed and collected under a microscope.

### RNA-sequencing and analysis

Total RNAs were extracted from cells using Trizol Reagent. DNA digestion was performed after extracting RNA by DNase I. A260/A280 absorbance was then measured to detect the quality of the RNA, and 1.5% agarose gel electrophoresis was used to detect the integrity of the RNA. Then, RNA sequencing library was established with KCTM Stranded mRNA Library Prep Kit for Illumina® according to the manufacturer’s instructions. Qualified reads of the obtained gene sequences were screened by Trimmomatic (version 0.36) and mapped to the corresponding genome. Reads in the exon region of each gene were counted, and the genes with statistical differences were screened by edgeR software package (version 3.12.1). Finally, Gene Ontology and KEGG analysis were used for gene set enrichment analysis.

### Transmission electron microscope (TEM) photograph of cell mitochondria

After treatment, the DLBCL cells were immobilized with transmission electron microscope (TEM) fixative for 2 h and then embedded with 1% agarose solution. After fixation with 1% OsO_4_ for 2 h and dehydrated at room temperature, samples were then permeated in the embedding plates and kept overnight at 37 °C. After drying the embedded plates, the resin blocks were cut into 60–80 nm slices by an ultra-thin microtome, and then stained with 2% uranium acetate-saturated alcohol solution. The morphology of cytoplasmic mitochondria was observed under the TEM.

### Mitochondria membrane potential assay

Cells were seeded in a 6-well plate and were treated as indicated for 48 h. Then the cells were collected and washed with PBS and incubated with Tetramethylrhodamine Ethyl Ester (TMRE) solution for 30 min at 37 °C. After centrifugation and washing, the cells were observed under a fluorescence microscope (Olympus).

### ROS assay

After drug treatment, the cells were centrifuged and incubated with DCFH-DA probe at 37 °C for 30 min. Then the cells were washed with serum-free medium to remove excessive probe. The changes of ROS level were observed under a fluorescence microscope (Olympus) and photographed.

### Analysis of MDA

After treatment, the cells were collected, washed with PBS, and ultrasonically broken with Cell lysis buffer (Beyotime). Then the protein concentration was determined by BCA kit and the remaining amount of protein lysate was centrifuged, added to the MDA working solution, and boiled at 100 °C for 15 min. After centrifugation, the absorbance was measured via Microplate Reader at 532 nm.

### Analysis of GSH

The GSH was measured with Micro GSH Assay Kit (Nanjing Jiancheng Bioengineering Institute) as indicated. Briefly, the cells or tissues were collected, the reagent 1 was added for fragmentation and centrifuged at 4 °C for 10 min. Then the supernatant was kept, and the reagents 2 and 3 were added for the color reaction. After 5 min, the absorbance was measured by Microplate Reader at 405 nm.

### Analysis of LPO

After drug treatment, the cells were collected and rinsed twice with PBS. Then the cells were incubated with 500 µL of diluted BODIPY 581/591 C11 probe (2 μM) for 30 min at 37 °C protected from light. The probe was then removed by centrifugation and rinsed twice with PBS. Finally, the LPO level was observed and photographed using an inverted fluorescence microscope.

### Ethics statement

The animal study was reviewed and approved by Southwest University Institutional Animal Care and Use Committee (IACUC-20231113-01). The human tissue samples were acquired from biopsies of DLBCL patients with informed consent. The experimental protocol was approved by the local ethics committee in Chongqing University Cancer Hospital in accordance with the Declaration of Helsinki (CZLS2023085-A-100).

## Results

### Ran strongly related to oncogenic USP13 in DLBCL

**Fig. 1:**
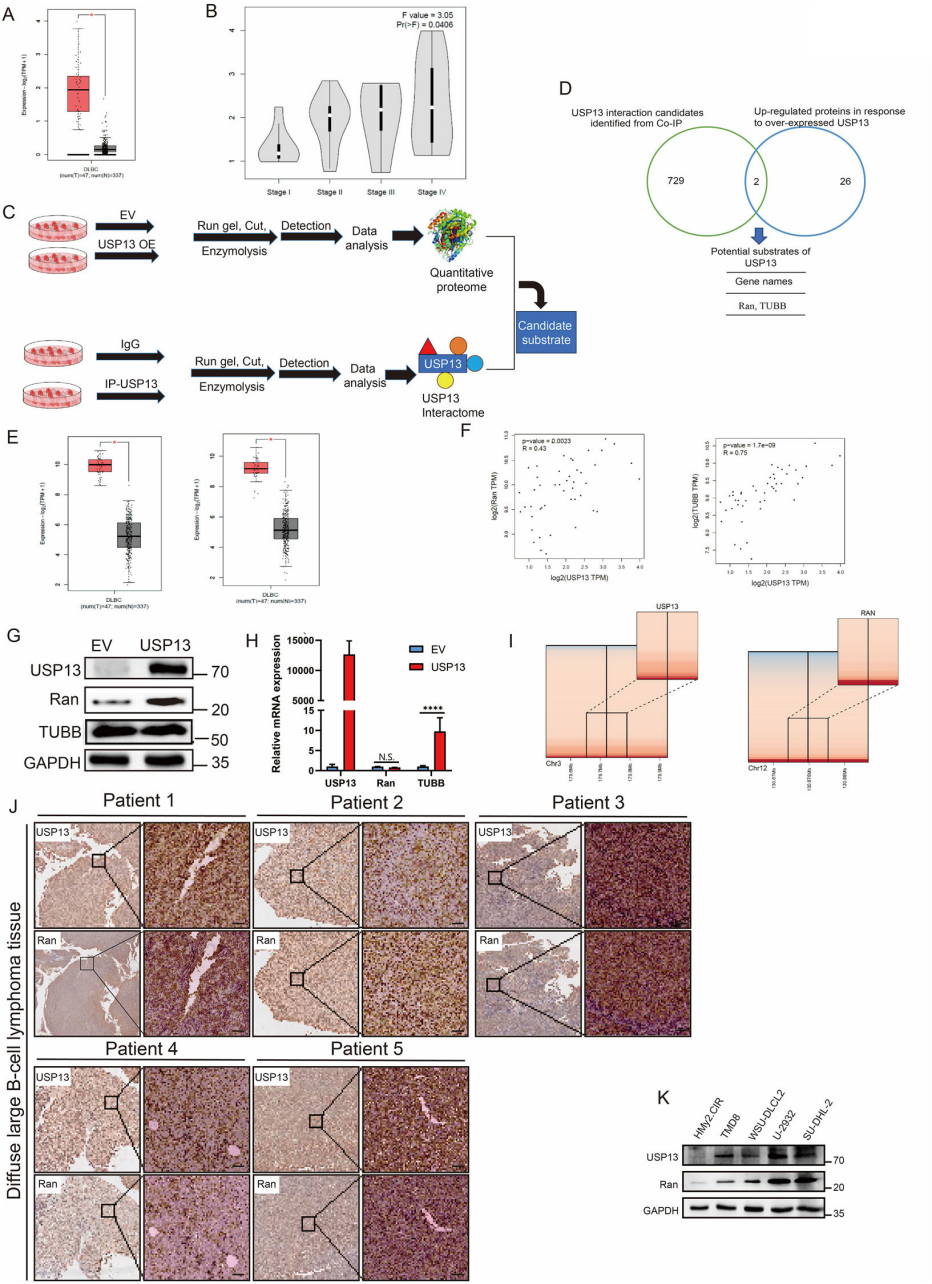
Identification of Ran as a candidate substrate of USP13. **A** Gene expression profiling (GEP) data showing USP13 expression in DLBCL: 47 patient samples and 337 normal control tissues. The figure was plotted from TCGA and GEPIA datasets. **B** The expression of USP13 in different DLBCL stages, and the figure was plotted from TCGA and GEPIA datasets. **C** Schematic diagram showing the process of USP13 substrate identification using quantitative proteomics by LC-MS/MS system. **D** Venn diagram showing the number of up-regulated proteins identified with USP13 overexpression (blue) and proteins of USP13 interactome identified in Co-IP process (green). The chart below showed the overlapped proteins. **E** GEP data showing Ran (left) and TUBB (right) expression in DLBCL: 47 patient samples and 337 normal control tissues. The figure was plotted from TCGA and GEPIA datasets. **F** The correlation analysis of USP13 and Ran (left, R = 0.43, *P* = 0.0023) or USP13 and TUBB (right, R = 0.75, *P* = 1.7e-09) in DLBCL samples plotted from TCGA and GEPIA datasets. **G** Immunoblots of the lysates from HEK293T transfected with empty vector (EV) and GFP-USP13 for 48 h. **H** Quantitative PCR analysis of USP13, Ran, and TUBB mRNA levels in HEK293T cells upon USP13 overexpression (N.S. not significant; ****P < 0.0001). **I** Copy number variation analysis of USP13 and Ran in TCGA DLBCL samples (DLBC cohort) with genomic deletion in blue and amplification in red. **J** Representative images for immunohistochemical staining of USP13 and Ran in human DLBCL tissue (scale bar: 50 μm). **K** Immunoblots for indicated proteins of lysates from normal B lymphocytes and a panel of DLBCL cell lines. *N* = 3 individual experiments and representative images are shown.

We assessed the significance of a range of DLBCL USPs through TCGA and GEPIA datasets and noted that USP13 was overexpressed in DLBCL (Fig. 1A) and elevated as the disease deteriorated (Fig. 1B) [22]. Further analysis showed that USP13 knockdown arrested DLBCL cell survival (Supplementary Fig. 1A, B) and genetic or pharmacological manipulation of USP13 markedly affected DLBCL cell migration (Supplementary Fig. 1C, D), indicating the prominent role of USP13 in DLBCL tumorigenesis. Given the property of USP13 as a DUB, we hypothesized that USP13 might mediate DLBCL pathogenesis via stabilizing downstream oncoproteins. Therefore, we transfected HEK293T cells and detected USP13 interactome and proteome with USP13 overexpression through LC-MS/MS analysis (Fig. 1C). Then, we overlapped proteins interacted with USP13 and proteins upregulated with USP13 overexpression, and identified Ran and TUBB related to USP13-mediated deubiquitination (Fig. 1D). Further analysis revealed that both Ran and TUBB were overexpressed in DLBCL patient samples (Fig. 1E). TUBB was positively and significantly correlated with USP13 at mRNA level (R = 0.75, *P* = 1.7e-09), while Ran mRNA was weakly correlated with USP13 (R = 0.43, *P* = 0.0023) (Fig. 1F). To verify the effect of USP13 on Ran and TUBB, we overexpressed USP13 and found that USP13 stabilized the Ran protein with mRNA intact, whereas TUBB demonstrated opposite and unaccountable trend (Fig. 1G, H), suggesting the role of Ran as potential USP13 substrate. Next, copy number analysis of TCGA DLBCL samples indicated that both USP13 and Ran were genetically amplified in some DLBCL cases (Figure I), suggesting a concomitant possibility of genetic correlation between USP13 and Ran. The translational level correlation was further confirmed by co-localization of USP13 and Ran in DLBCL patient sample staining (Fig. 1J). Meanwhile, both USP13 and Ran were scarcely expressed in normal B cells but overexpressed and correlated in DLBCL cell lines at protein level (Fig. 1K). Altogether, these results indicated that USP13 and Ran were overexpressed and genetically and translationally correlated in DLBCL, and overexpression of USP13 stabilized Ran protein.

### USP13 interacted with Ran for enzymatic stabilization

**Fig. 2:**
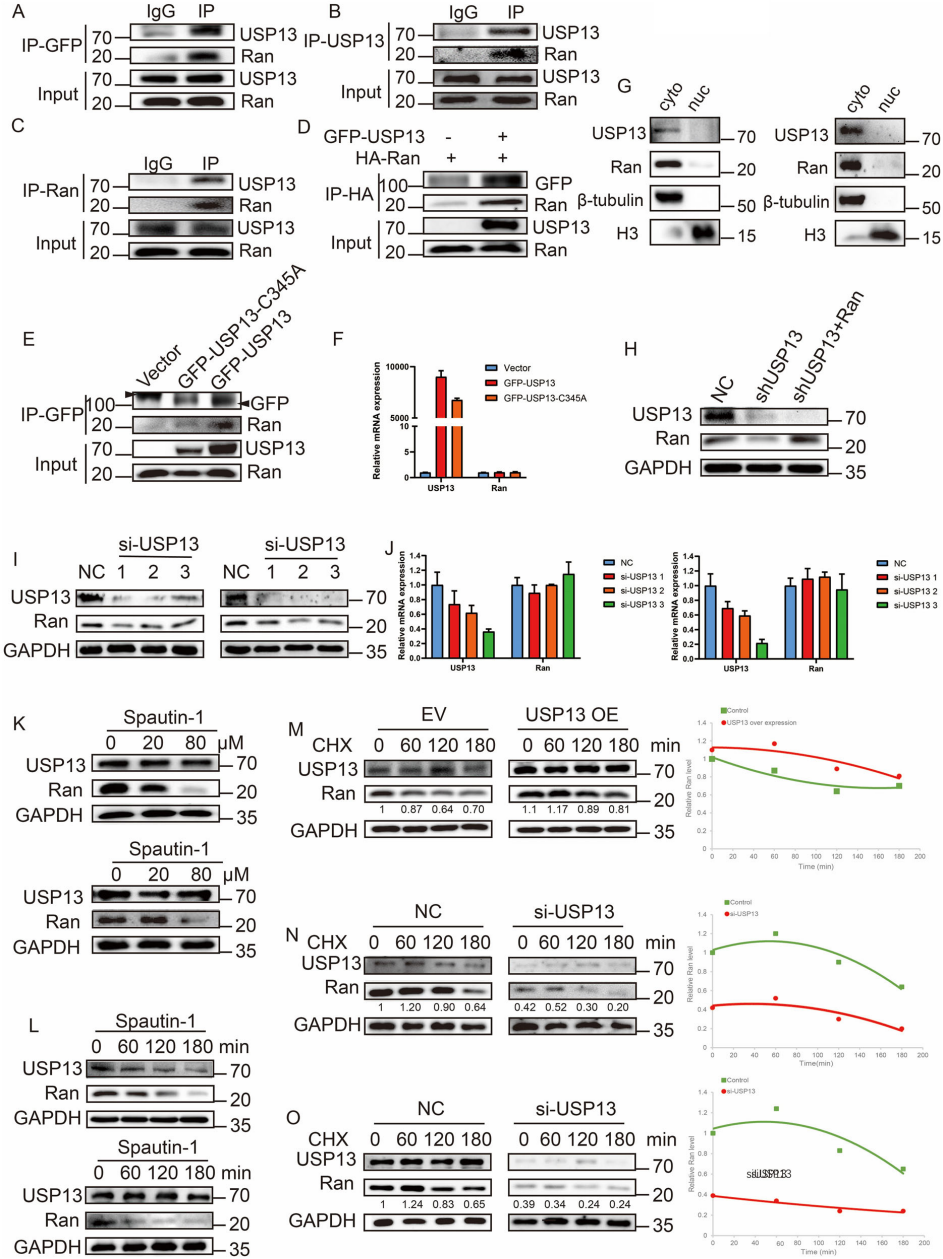
USP13 interacts with Ran and promotes Ran stability. **A** GFP-USP13 was transfected into HEK293T cells. USP13 was immunoprecipitated using anti-GFP antibody. **B** Co-IP showing interaction between USP13 and Ran with USP13 pull-down in OCI-LY3 cells. **C** Co-IP showing interaction between Ran and USP13 with Ran pull-down in SU-DHL-2 cells. **D** Co-IP showing change of interaction between USP13 and Ran with or without USP13 overexpression. **E** GFP-USP13 or its enzymatically-null mutant GFP-USP13 C345A was transfected into HEK293T cells and immunoprecipitated using anti-GFP antibody. **F** Quantitative PCR analysis of USP13 and Ran mRNA levels in HEK293T cells with indicated transfections. **G** Nuclear-cytoplasmic fractionation in U2932 (left) and WSU-DLCL2 (right) cell lines with immunoblots for indicated proteins. **H** Immunoblots for the overexpression of Ran in USP13-depeleted HEK293T cells. **I** Ran protein level change upon USP13 knockdown using siRNA in SU-DHL-2 (left) and U-2932 (right) cells. **J** Ran mRNA level change upon USP13 knockdown using siRNA in SU-DHL-2 (left) and U-2932 (right) cells. DLBCL cells were harvested for RNA extraction 24 h after knockdown. **K** Spautin-1 was given at indicated dosage and immunoblots were done using indicated antibodies in SU-DHL-2 (upper) and OCI-LY3 (lower) cells. **L** Spautin-1 was given at indicated slots and immunoblots were done using antibodies against indicated proteins in SU-DHL-2 (upper) and OCI-LY1 (lower) cells. **M** HEK293T cells with empty vector (EV) transfection or USP13 overexpression (OE) were treated with cycloheximide (CHX, 10 µg/mL) for different slots, and immunoblotted with antibodies against indicated proteins (left). Quantification of Ran protein levels were plotted normalized to GAPDH at different slots (right). **N**, **O** OCI-LY7 (**N**) or OCI-LY1 (**O**) were transfected with USP13 siRNAs, treated with CHX (10 µg/mL) for different slots, and immunoblotted with antibodies against indicated proteins (Left). Quantification of Ran protein levels were plotted normalized to GAPDH at different slots (right). *N* = 3 individual experiments and representative images are shown.

To explore the relationship between USP13 and Ran, we immunoprecipitated USP13 to check Ran expression and vice versa, and interactions between USP13 and Ran were captured in both DLBCL and HEK293T cells (Fig. 2A–D, Supplementary Fig. 2A). Compared with USP13 wild type (WT), the enzymatically-null mutant displayed decreased interaction with Ran (Fig. 2E) and compromised Ran stabilization (Supplementary Fig. 2B) with Ran mRNA unaffected (Fig. 2F). The association between USP13 and Ran was also modeled by Alphafold3 with high possibility of interaction predicted (Supplementary Fig. 2C) [23]. Both USP13 and Ran are located in the cytoplasm rather than the nucleus or mitochondria (Fig. 2G, Supplementary Fig. 2D). Knockdown of USP13 destabilized Ran protein without affecting its mRNA, and the simultaneous Ran overexpression cannot rescue USP13 knockdown, hinting Ran as a downstream substrate of USP13 (Fig. 2H–J). Then, we treated DLBCL cells with USP13 enzymatic inhibitor Spautin-1, and observed a decrease of Ran level in a dose- and time-dependent manner (Fig. 2K, L). With the treatment of the de-novo protein synthesis inhibitor, cycloheximide, overexpression of USP13 led to a delay in the reduction of Ran level (Fig. 2M), and conversely, knockdown of USP13 hastened the decrease of Ran levels (Fig. 2N, O). All these data suggested that USP13 and Ran co-localized and interacted with each other, and USP13 stabilized Ran as a DUB.

### USP13 initiated Ran poly-ubiquitination

**Fig. 3:**
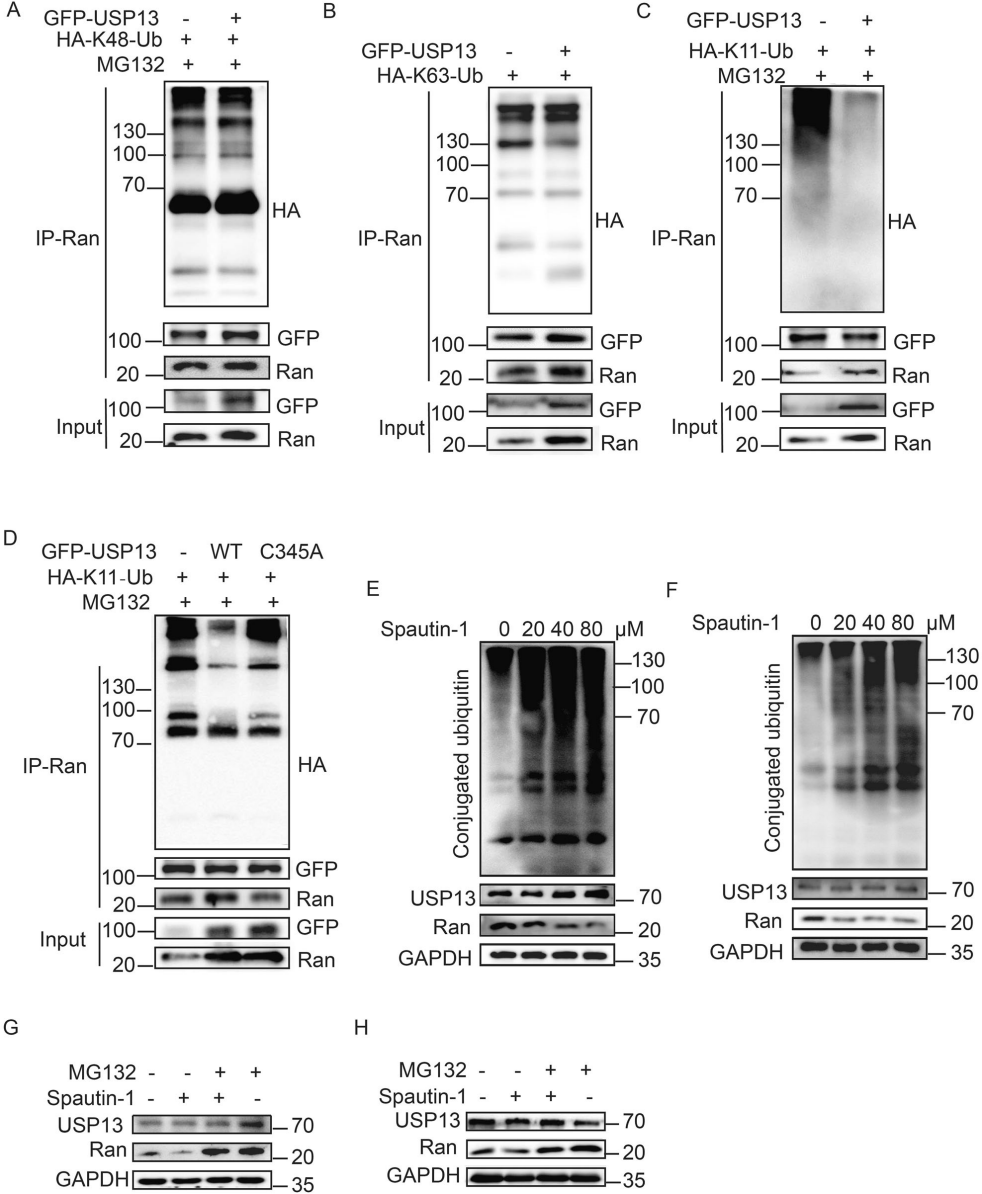
USP13 initiates K11-linked polyubiquitination of Ran. **A**–**C** Change of Ran K48- (**A**), K63- (**B**) or K11- (**C**) linked polyubiquitination with USP13 overexpression in HEK293T cells. **A**, **C** MG132 was given at 5 µM for 6 h of treatment 48 h after transfection. **D** Change of K11-linked polyubiquitination of Ran with GFP-USP13 or GFP-USP13-C345A mutant overexpression in HEK293T cells. **E**, **F** Change of conjugated ubiquitin and Ran level with spautin-1 treatment at indicated dosage in OCI-LY7 (**E**) and TMD8 (**F**) cells. **G**, **H** Immunoblots showing change of USP13 and RAN level upon spautin-1 (20 μM) and MG132 (10 μM) treatment for 6 h in SU-DHL-2 (**G**) and TMD8 (**H**) cells. *N* = 3 individual experiments and representative images are shown.

In line with the findings above, we went on to examine whether USP13 triggered Ran ubiquitination and the topologies of ubiquitin chain. As illustrated, USP13 overexpression did not affect K48-based ubiquitination of Ran (Fig. 3A), slightly inhibited its K63 ubiquitination (Fig. 3B), but conspicuously suppressed K11-linked Ran ubiquitination (Fig. 3C). K11-linked chains are the third most abundant polyubiquitination in cells and typically function in mediating protein degradation as well as cell cycle progression and differentiation [24, 25]. The declined K11 Ran ubiquitination could be rescued by USP13 enzymatically-null C345A mutant transfection (Fig. 3D). As expected, inhibition of USP13 activity via Spautin-1 treatment induced Ran ubiquitination in DLBCL cells in addition to repressing Ran level (Fig. 3E, F). Moreover, treatment with protease inhibitor MG132 completely abrogated Spautin-1-induced Ran destabilization (Fig. 3G, H), indicating that USP13 stabilized Ran via blocking proteasome-mediated degradation. These results demonstrated that USP13 stabilized Ran by promoting K11-linked polyubiquitination and blocking proteasome-mediated degradation.

### Spautin-1 impeded DLBCL tumorigenesis via intervening Notch and NF-κB

**Fig. 4:**
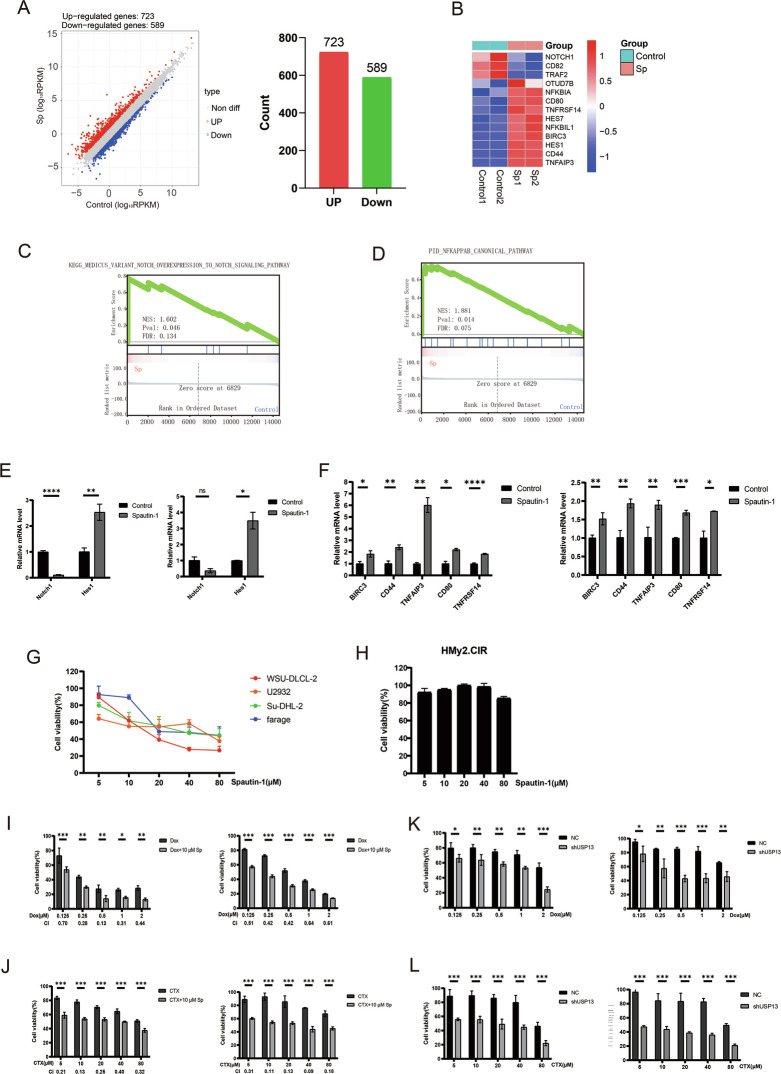
Spautin-1 inhibits DLBCL cell survival and synergizes with Doxorubicin (Dox) or Cyclophosphamide (CTX). **A** TMD8 cells treated with Spautin-1 or solvent control for 36 h were subjected to RNA-seq. The number of differentially-expressed genes were indicated in volcano plot (left) or bar chart (right). **B** Heatmap indicating differentially-expressed genes in TMD8 cells treated with Spautin-1 for 36 h compared with solvent control. **C**, **D** Gene set enrichment analysis identified gene sets describing enrichment of Notch (left) and NF-κB (right) pathway targets upon treatment with Spautin-1. **E** Quantitative Real-time PCR indicating expression of genes in Notch pathway in DLBCL cells treated with Spautin-1(10 μM) for 36 h in SU-DHL-2 (left) or TMD8 (right) cells. **F** Quantitative Real-time PCR indicating expression of genes in NF-κB pathway in DLBCL cells treated with Spautin-1(10 μM) for 36 h in SU-DHL-2 (left) or TMD8 (right) cells. **G**, **H** The survival rate of DLBCL cells (**G**) and normal lymphocytes (**H**) was measured by Cell Counting Kit-8 assay after 48 h treatment with Spautin-1 at indicated dosage. **G**, **H** Cell viability of DLBCL cells (**G**) or normal B lymphocytes (**H**) treated for 48 h with Spautin-1 at indicated doses. Cell Counting Kit-8 assay was used to measure cell viability. **I** Cell viability of DLBCL cells treated for 48 h with the combination of Spautin-1(10 μM) and Dox of indicated dosage in WSU-DLCL-2 (left) or U2932 (right) cells. **J** Cell viability of DLBCL cells treated for 48 h with the combination of Spautin-1 (10 μM) and CTX of indicated dosage in WSU-DLCL-2 (left) or Farage (right) cells. **I**, **J** The CI values was calculated by CompuSyn software, and CI values were all less than 1. **K** Cell viability of USP13-knockdown DLBCL cells treated for 48 h with Dox of indicated dosage in WSU-DLCL-2 (left) or U2932 (right) cells. **L** Cell viability of USP13-knockdown DLBCL cells treated for 48 h with CTX of the indicated dosage in WSU-DLCL-2 (left) or U2932 (right) cells. *P < 0.05, **P < 0.01, ***P < 0.001, ****P < 0.0001. N = 3 individual experiments.

To interrogate USP13 downstream pathways that may mediate DLBCL pathogenesis, we performed RNA-seq following treatment of the DLBCL cell line with Spautin-1. The results revealed 723 upregulated genes and 589 downregulated genes (Fig. 4A), with Notch and canonical NF-κB pathways most enriched as visualized by heatmap (Fig. 4B) and GSEA analysis (Fig. 4C, D). Alterations of gene transcription in Notch (NOTCH1 and HES1) and NF-κB (BIRC3, CD44, TNFAIP3, CD80 and TNFRSF14) pathways were then validated (Fig. 4E, F). The regulatory effect of USP13 knockdown on the transcript levels of these genes was consistent with Spautin-1 treatment (Supplementary Fig. 3A, B).

### Spautin-1 synergized with Dox and CTX

A panel of DLBCL cell lines and a normal B lymphocyte line were subjected to Spautin-1 treatment. As illustrated, Spautin-1 treatment effectively repressed survival of DLBCL cells with the best IC50 of around 15 μM (Fig. 4G). In contrast, this compound seldom affected survival of normal B lymphocytes (Fig. 4H). Given Dox and CTX as the conventional and first-line therapeutics to treat DLBCL, which also functioned via Notch and NF-κB signaling pathways (Supplementary Fig. 4) [26], we hypothesized that Spautin-1 might synergize with Dox or CTX. Indeed, Spautin-1 treatment in DLBCL was synergistic with Dox or CTX, as indicated by the combination index, compared to treatment with Dox or CTX alone (Fig. 4I, J). Similarly, knockdown of USP13 accentuated the effects of Dox or CTX (Fig. 4K, L). All the above results showed that Spautin-1 arrested DLBCL cell survival without affecting normal lymphocytes, and synergized with Dox or CTX, possibly due to intervention with Notch and NF-κB.

### Spautin-1 induced ferroptosis in combination with Dox or CTX

**Fig. 5:**
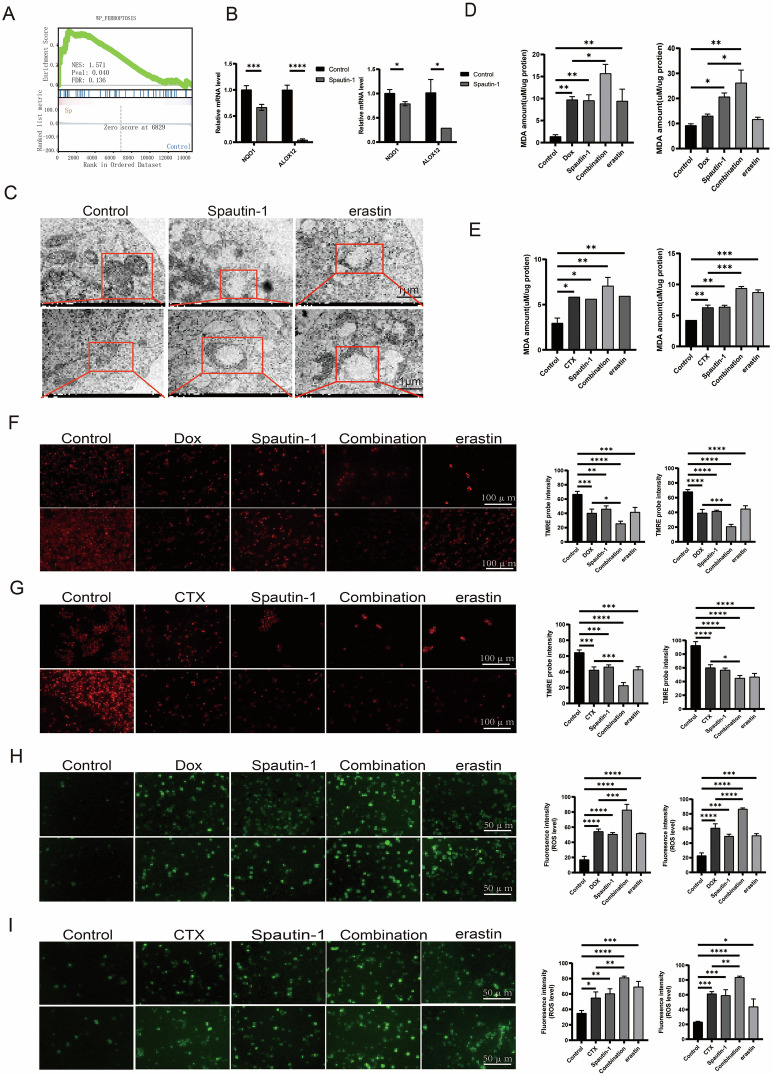
Spautin-1 induces DLBCL cell ferroptosis in combination with Dox or CTX. **A** Gene set enrichment analysis identified a gene set describing ferroptosis targets enriched upon treatment with Spautin-1 in TMD8 cells. **B** Quantitative Real-time PCR indicating expression of NQO1 and ALOX12 treated with Spautin-1 (10 μM) for 36 h in TMD8 (left) and SU-DHL-2 (right) cells. **C** The morphology of mitochondria in TMD8 (upper) or SU-DHL-2 (lower) cells treated as control, with Spautin-1 (40 μM) or with erastin for 48 h visualized by transmission electron microscopy. The scale was 1 μm. **D** The Lipid peroxide level was examined after treatment with Dox (1 μM), Spautin-1(10 μM) or in combination or erastin (2 μM) by using Lipid Peroxidation MDA Assay Kit in WSU-DLCL-2 (left) or U2932 (right) cells. **E** The Lipid peroxide level was examined after treatment with CTX (20 μM), Spautin-1(10 μM) or in combination or erastin (2 μM) by using Lipid Peroxidation MDA Assay Kit in WSU-DLCL-2 (left) or U2932 (right) cells. **F** The mitochondrial membrane potential was quantified by inverted fluorescence microscope using TMRE probe in SU-DHL-2 (upper) or TMD8 (lower) cells after treatment with Dox (1 μM), Spautin-1(10 μM) or in combination or erastin (2 μM) for 48 h. The fluorescence intensity was visualized as bar chart (left: SU-DHL-2; right: TMD8). **G** The mitochondrial membrane potential was quantified by inverted fluorescence microscope using TMRE probe in SU-DHL-2 (upper) or TMD8 (lower) cells after treatment with CTX (20 μM), Spautin-1(10 μM) or in combination or erastin (2 μM) for 48 h. The fluorescence intensity was visualized as bar chart (left: SU-DHL-2; right: TMD8). **H** The ROS level was quantified by inverted fluorescence microscope in DLBCL cells after treatment with Dox (1 μM), Spautin-1(10 μM), or in combination or erastin (2 μM) using DCFH-DA probe in U2932 (upper) or WSU-DLCL-2 (lower) cells. The fluorescence intensity was visualized as bar chart (left: U2932; right: WSU-DLCL-2). **I** The ROS level was quantified by inverted fluorescence microscope in DLBCL cells after treatment with CTX (20 μM), Spautin-1(10 μM) or in combination or erastin (2 μM) using DCFH-DA probe in U2932 (upper) or WSU-DLCL-2 (lower) cells. The fluorescence intensity was visualized as bar chart (left: U2932; right: WSU-DLCL-2).

A further in-depth analysis of the abovementioned RNA-seq data identified ferroptosis as a critical event upon Spautin-1 treatment in DLBCL cells as characterized by GSEA analysis (Fig. 5A). Upon addition of Spautin-1, the ferroptosis antagonist NQO1 and ALOX12 exhibited sharp decrease (Fig. 5B). Accordingly, upon USP13 knockdown the transcript levels of NQO1 and ALOX12 drastically reduced (Supplementary Fig. 5A). In addition, Fer-1, an inhibitor of ferroptosis, significantly rescued Spautin-1-induced cell death in DLBCL cells (Supplementary Fig. 5B). As mitochondria is one of the key organelles targeted during ferroptosis, Spautin-1 treatment prompted morphological changes of mitochondria including reduced or absent cristae and rupture of outer membrane similar to the widely-acknowledged ferroptosis inducer erastin (Fig. 5C). During ferroptosis, long-chain poly-unsaturated fatty acids are incorporated into phospholipid membranes to trigger lipid peroxidation reactions which is the ultimate event before ferroptosis [27]. As shown, Spautin-1 synergized with Dox and CTX to upregulate MDA level indicating the induction of peroxidation chain reactions (Fig. 5D, E). In combination with Dox or CTX, Spautin-1 further alleviated mitochondria membrane potential in line with erastin, suggesting mitochondria damage (Fig. 5F, G). Moreover, in ferroptosis the oxidative stress is increased and GSH is decreased in related to dysfunctional antioxidant defense, with LPO upregulation as the ferroptosis execution step [27]. Correspondingly, Spautin-1 treatment in DLBCL cells enhanced ROS, LPO, and suppressed GSH level in synergy with Dox and CTX (Fig. 5H, I, Supplementary Fig. 5C, D, Supplementary Fig. 6A, B). And knockdown of USP13 promoted Dox and CTX-induced elevation of ROS and MDA levels in DLBCL cells (Supplementary fig. 7A–D). These results uncovered that genetic ablation or pharmacologic inhibition of USP13 hampered mitochondria function and metabolism to induce ferroptosis in DLBCL, and these effects well combined with Dox or CTX treatment.

### Spautin-1 synergized with Dox or CTX to induce ferroptosis and suppressed tumor growth

**Fig. 6:**
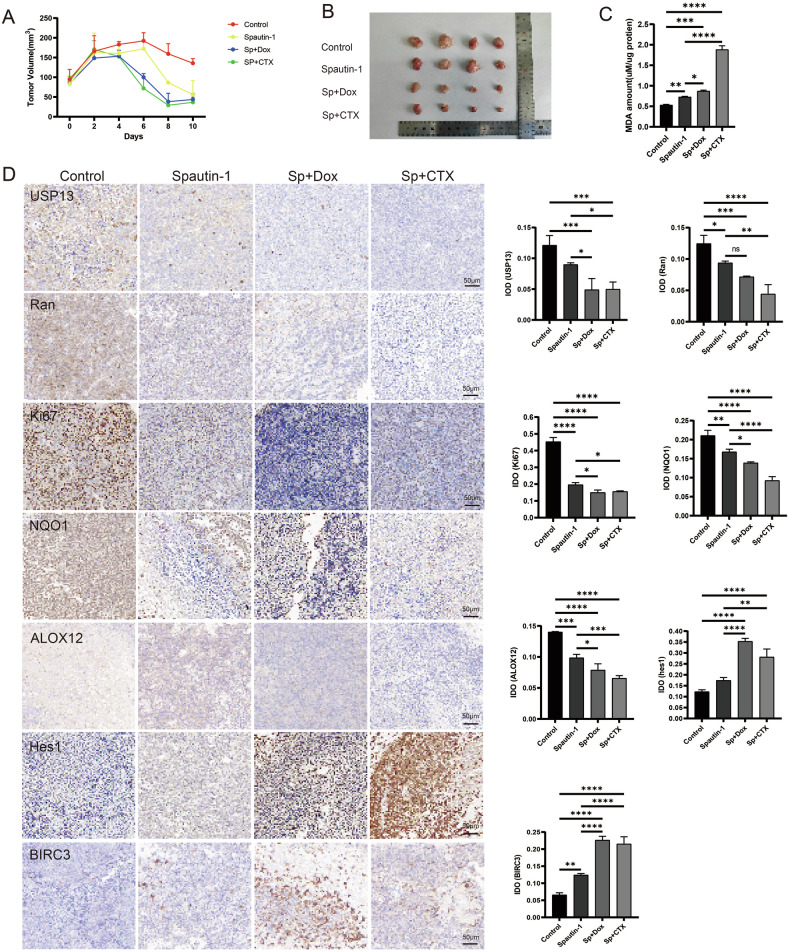
Spautin-1 synergizes with Dox or CTX to inhibit the growth of xenografted DLBCL tumor. **A**, **B** Moded NOD-SCID mice were administered as vehicle control, Spautin-1 (40 mg/kg /two days, i.p), Spautin-1+Dox (2 mg/kg/four days, i.g) or Spautin-1 + CTX (25 mg/kg/4 days, i.g) for two weeks. The tumor volume was measured every two days to draw the transplanted tumor growth curve (**A**). After the mice were sacrificed, the tumors were removed from the mice and photographed (**B**). **C** Lipid peroxide levels in mouse tumor tissue were quantified by Lipid Peroxidation MDA Assay Kit. **D** Immunohistochemical staining of USP13, Ran, Ki67, NQO1, ALOX12, Hes1 and BIRC3 of transplanted DLBCL tumor tissue. Representative images were shown (left). The staining was also quantified by ImageJ (right).

Subsequently, we examined the in-vivo effects of Spautin-1 or its combination with Dox or CTX in DLBCL malignancy. Subcutaneously-implanted mouse models were treated as control, Spautin-1 or Spautin-1 with Dox or CTX. We observed reduction of tumor volume in Spautin-1-treated group compared with the control and further reduction upon the co-treatment with Dox or CTX (Fig. 6A, B). The induction to ferroptosis with single treatment or combination in mouse models was confirmed by the further increase of MDA levels in lipid peroxidation (Fig. 6C) and reduction of GSH levels (Supplementary Fig. 6C) with chemo-combination. Accordingly, the dissected specimens from tumor tissues demonstrated further reduction of USP13 and Ran expression as well as the proliferative marker Ki-67, the ferroptosis mediator NQO1 and ALOX12, and increase in the expression of HES1, a Notch pathway marker, and BIRC3, a marker in the NF-κB pathway in combination groups compared with the single treatment group (Fig. 6D). Drug treatment with the indicated doses had minimal effect on the morphology of the main organs including heart, liver, spleen, lung and kidney (Supplementary fig. 8), therefore may not yield apparent toxicity. This suggested the combination between Spautin-1 and Dox or CTX synergized via USP13-Ran signaling, induced ferroptosis, and arrested tumor growth via downstream Notch and NF-κB pathways.

## Discussion

In this study, we have identified Ran that interacts with and is deubiquitinated by USP13 through MS-based screening, and pharmacological inhibition of USP13 by Spautin-1 synergizes with Dox or CTX to induce ferroptosis in DLBCL possibly via the USP13-Ran signaling. Moreover, USP13 and Ran are genetically amplified and colocalized in some DLBCL cases and ubiquitously overexpressed in DLBCL cell lines. Therefore, our results demonstrate that Ran is a novel substrate of USP13, and Spautin-1 might be a new therapeutic approach to complement conventional treatment of DLBCL.

Several other studies have implied the role of USP13 by mediating the turnover of different oncogenes or tumor suppressors. However, the pathogenic function of USP13 seems to be context-dependent. For instance, USP13 deubiquitinated c-MYC in glioblastoma and lung squamous cell carcinoma to promote disease progression [12, 21]; USP13 oncogenically stabilized MCL-1 in ovarian and lung cancer and de-sensitized tumor cells to BH3 mimetic inhibitors [13]. In contrast, USP13 may act as a PTEN DUB, leading to suppressed AKT phosphorylation, glycolysis, and tumor cell proliferation in breast cancer [14]. Here, the role of USP13 in DLBCL has never been characterized before, and here we provide multiple lines of evidence indicating that tumorigenic USP13 deubiquitinates and stabilizes Ran in this malignancy. First, USP13 and Ran were overexpressed in TCGA dataset and DLBCL cell lines, and colocalized in DLBCL patient samples. Furthermore, USP13 promoted Ran protein stability without affecting its mRNA level. Finally, Alphafold3 modeling and wet bench validation indicated USP13 interacted with Ran, and USP13 attenuated K11- and K63-linked polyubiquitination of Ran. These findings confirm the function of USP13 specifically in DLBCL and enrich the connotation of USP13 as a DUB.

Ran is physiologically involved in the regulation of nuclear transport and microtubule spindle assembly during cell division and pathologically involved in tumor proliferation, cell cycle perturbation, and metastasis [28, 29]. Although the tumorigenic role of Ran has been elucidated in multiple contexts, pharmacological approaches of Ran intervention are not yet available clinically. This study fills in the gap by clarifying the USP13-Ran regulating axis, which hints destruction of Ran protein via enzymatic inhibition of USP13 activity by Spautin-1 treatment. The current study also extends the understanding of the regulation of Ran stability. Previous studies have uncovered that the stability of Ran was mostly governed by sumoylation [30] and mediated by long non-coding RNAs [31]. And here we show that USP13 dictates Ran stability through deubiquitination, and Spautin-1 treatment recovers Ran polyubiquitination level.

Dox and CTX are components of classical R-CHOP regimen and two of the first-line chemo-agents in DLBCL. Significant toxicities arising from Dox and CTX therefore requiring dose modifications in drug combinations [32]. Given that the resistance to Dox or CTX was partially mediated by Notch and NF-κB pathway nodes and that Spautin-1 treatment also signaled through these two pathways, we thereby combined Spautin-1 with Dox or CTX. Notably, within the combination group, no visible morphological alterations of organs including heart, liver, spleen, lung, and kidney were detected with significant reduction in tumor burden, indicating notable therapeutic effects with no obvious toxicities. This suggests the combination may be useful in DLBCL treatment.

Ferroptosis represents an iron-dependent and peroxidation-driven programmed cell death. Accompanied by morphological changes of mitochondria, induction of oxidative stress, repression of GSH and finally, lipid peroxidation, ferroptosis is an avenue for chemo-agents to treat cancer [18]. Our study has identified a significant enrichment of ferroptosis-related genes, and reduced cristae and vacuolation of the mitochondria upon Spautin-1 treatment. The induction of ferroptosis was also one of the main mechanisms underlying the synergy between Spautin-1 and Dox or CTX, as the combinational treatment further hampered mitochondria integrity and GSH level as well as elevating ROS, MDA and LPO level resulting in lipid peroxidation. All the evidence suggests Spautin-1 effectively suppresses DLBCL cell survival and synergizes with Dox or CTX at least partly via ferroptosis induction.

In conclusion, our study has identified Ran as a novel substrate of the DUB USP13 in DLBCL. Pharmacological inhibition of USP13 by Spautin-1 diminishes Ran level and induces ferroptosis. At least partially due to the modulation of Notch and NF-κB pathway, Spautin-1 well synergizes with Dox or CTX with demonstrated efficacy and undetectable toxicities. Our study has therefore provided a rationale for devising clinical trials to combine Spautin-1 with R-CHOP regimen for DLBCL treatment.

## Supplementary information

Supplemental figures

WB original data

## Data Availability

Raw data of the LC–MS/MS analysis have already been uploaded to proteomeXchange Consortium via PRIDE (PXD056840, PXD056804). Raw data of the RNA-seq analysis has been uploaded to Gene Expression Omnibus (GSE280321).
