# Supplementary material for: USP13 dictates Ran turnover and vulnerability to ferroptosis in diffuse large B cell lymphoma (DLBCL)
Source: Cell Death Dis. 2025 Nov 28;16(1):870. doi: 10.1038/s41419-025-08207-6 (PMC12663190; doi:10.1038/s41419-025-08207-6)
Supplement: Supplementary file 1 — Supplemental figures [file 41419_2025_8207_MOESM1_ESM.docx]

**Supplemental figures**

**Supplemental Figure 1**. **USP13 is critical to DLBCL pathogenesis.** (A and B) Relative viability of SU-DHL-2 (A) or TMD8 (B) cells infected with lentivirus encoding short hairpin RNA targeting USP13 or negative control (NC). Cell Counting Kit-8 assay was used to measure cell viability. (C) Transwell assay indicating migration of Farage and OCI-LY3 cells with or without Spatutin-1 (80 μM) treatment. (D) Transwell assay indicating migration of SU-DHL-2 and TMD8 cells with or without USP13 depletion.

**Supplemental figure 2**. **Cytosolic USP13 interacts with Ran.** (A) Co-IP demonstrating intrinsic interaction between USP13 and Ran in SU-DHL-2 cells. (B) Change of Ran level with GFP-USP13 or GFP-USP13-C345A overexpression. (C) Diagram from AlphaFold server modeling protein–protein interaction between USP13 and Ran. (D) Immunoblots of indicated proteins in cytosol and mitochondria fractions in U-2932 (left) and WSU-DLCL2 (right) cells.


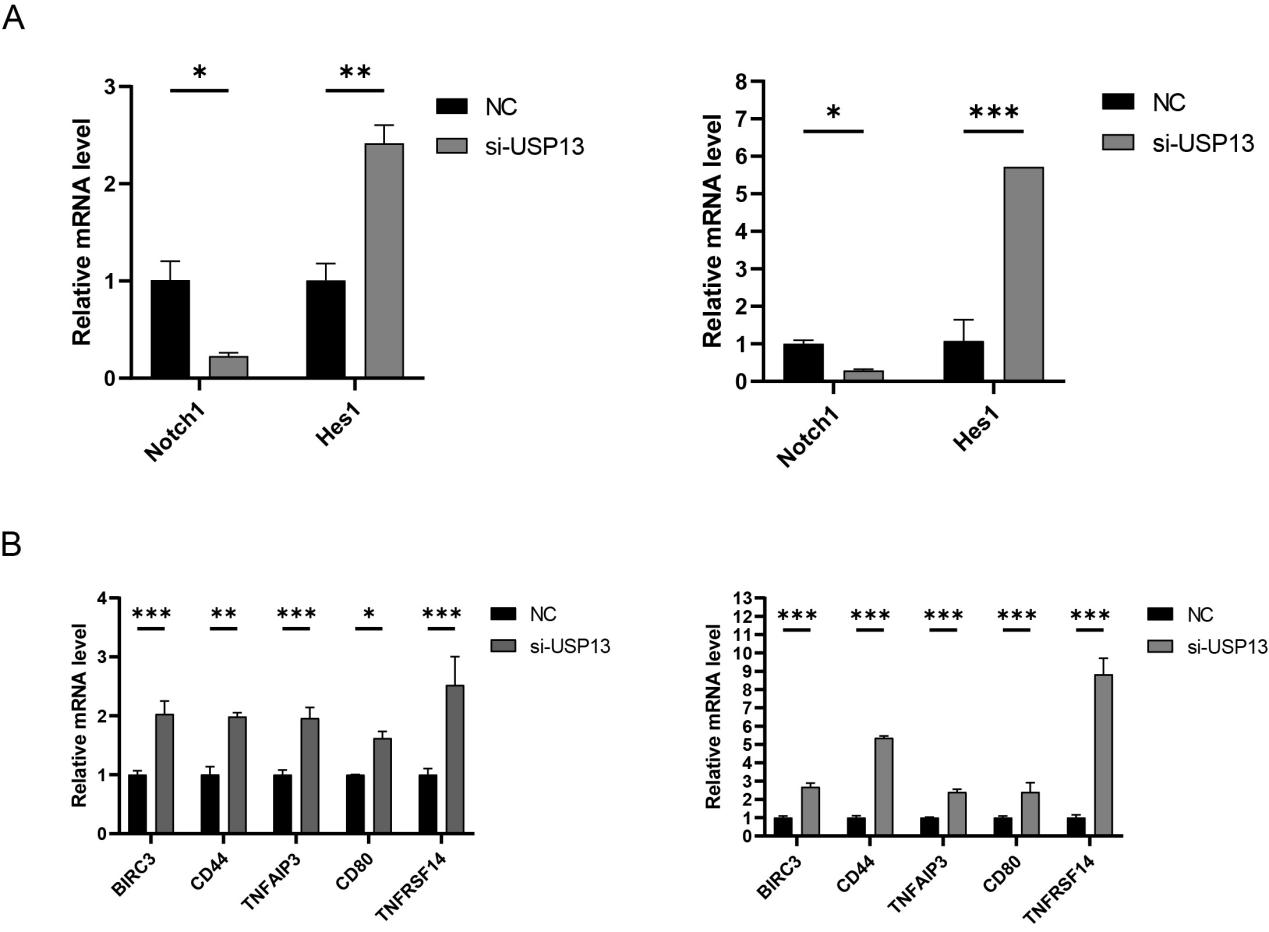


**Supplemental figure 3. Effect of knockdown of USP13 on transcript levels of Notch and NF-KB pathway related genes in DLBCL cells.** (A) USP13 knockdown in DLBCL cells was followed by Quantitative Real-time PCR to determine Notch pathway-related gene expression in SU-DHL-2 (left) and U2932 cells (right). (B) USP13 knockdown in DLBCL cells was followed by Quantitative Real-time PCR to determine NF-κB pathway-related gene expression in SU-DHL-2 (left) and U2932 cells (right).


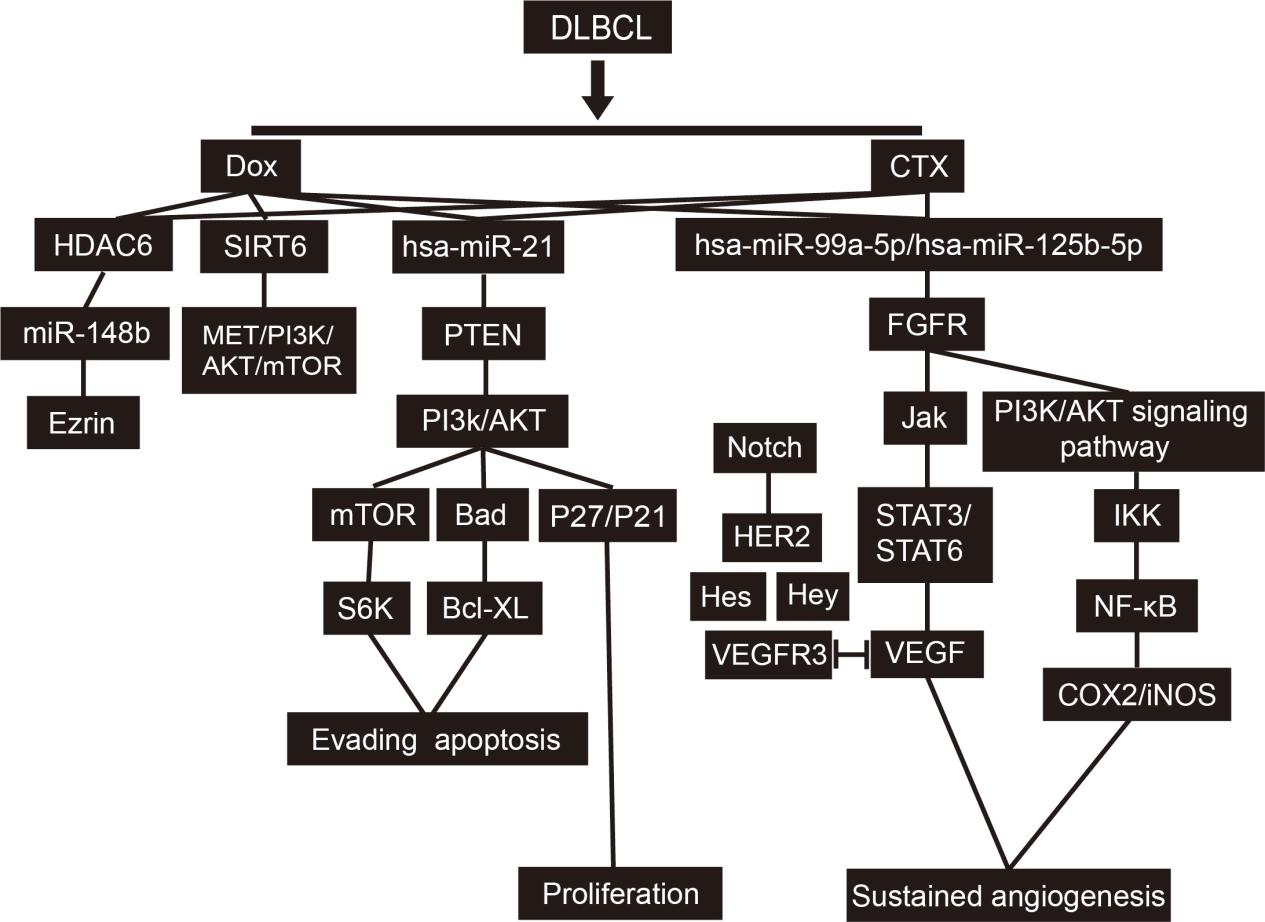
**Supplemental figure 4**. **Schematic diagrams illustrating regulations of sensitivity to Dox or CTX in DLBCL.** The raw data was drawn from the DRESIS database or from existing literature.


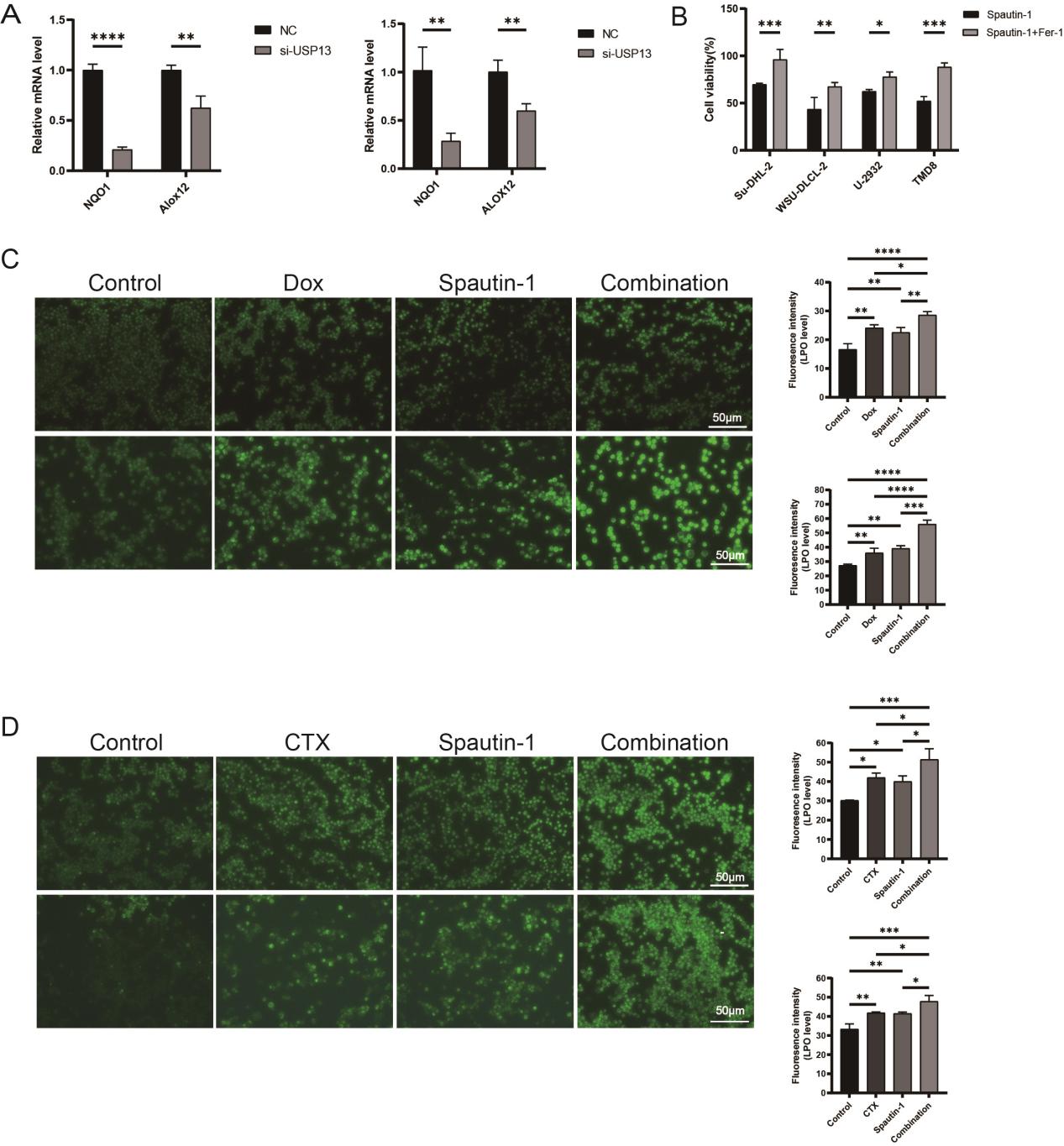


**Supplemental figure 5. Spautin-1 combined with Dox or CTX to execute ferroptosis.** (A) Knockdown of USP13 in DLBCL cells was followed by Quantitative Real-time PCR to determine ferroptosis pathway-related gene expression in U2932 (left) and SU-DHL-2 cells (right). (B) Cell viability of DLBCL cells treated for 48 h with Spautin-1 (10 μM) or a combination of Spautin-1 (10 μM) and Fer-1 (10 μM). (C) After treatment with Spautin-1(10 μM), Dox (1 μM) or a combination of both in DLBCL cells, LPO levels were measured by using the BODIPY 581/591 C11 probe in WSU-DLCL-2 (upper) and U2932 (lower) cells. The fluorescence intensity was visualized as bar chart (upper: WSU-DLCL-2; lower: U2932). (D) After treatment with Spautin-1 (10 μM), CTX (20 μM) or a combination of both in DLBCL cells, LPO levels were measured by using the BODIPY 581/591 C11 probe in WSU-DLCL-2 (upper) and U2932 (lower) cells. The fluorescence intensity was visualized as bar chart (upper: WSU-DLCL-2; lower: U2932).


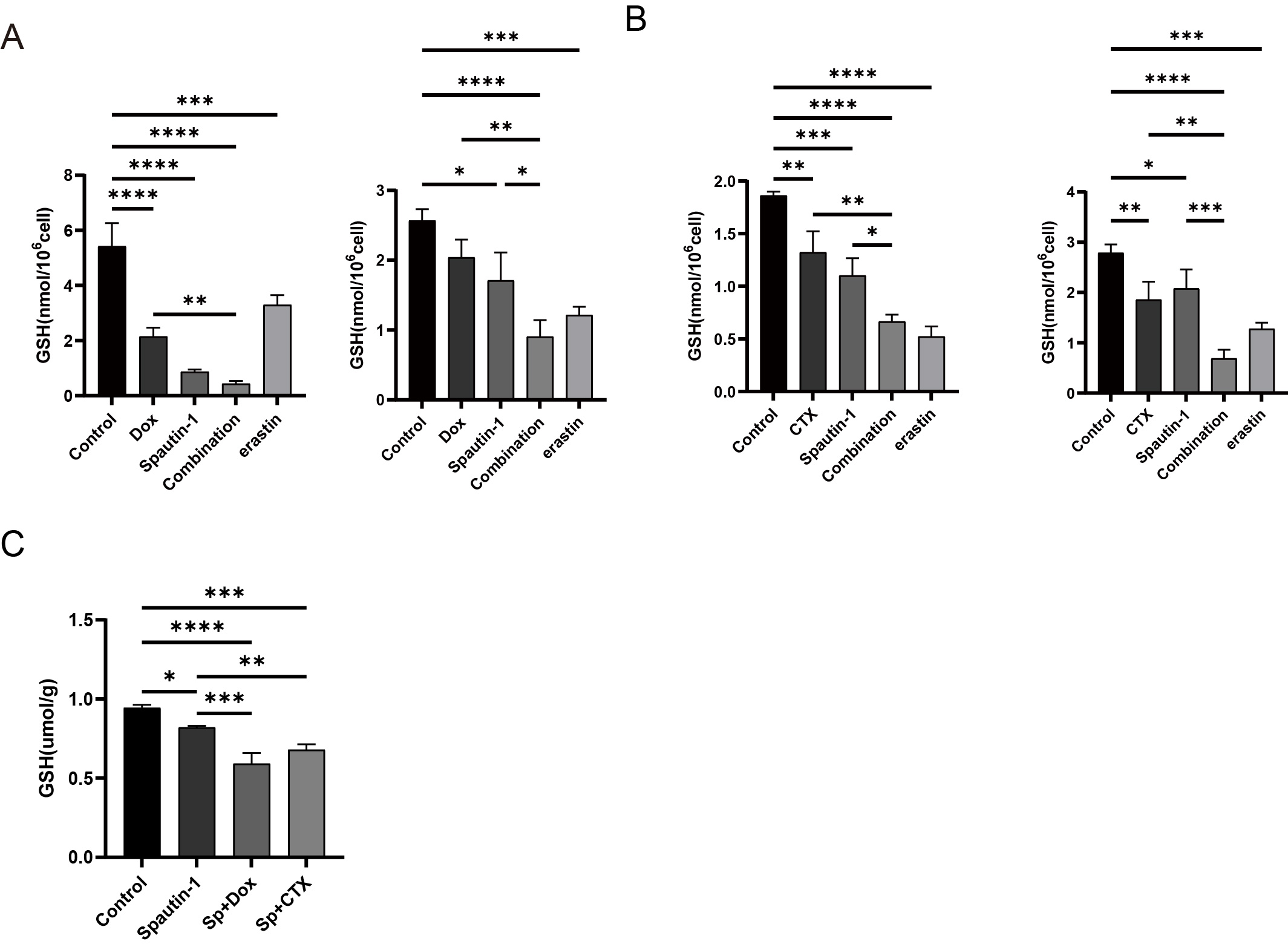


**Supplemental figure 6. Regulation of GSH levels in DLBCL cell lines and tumor tissues with Spautin-1 and Dox or CTX treatment in combination.** (A) DLBCL cells (left: WSU-DLBCL-2; right: SU-DHL-2) were treated with Dox (1 μM), Spautin-1(10 μM), Combination or erastin (2 μM) and the GSH level was quantified by Micro GSH Assay Kit. (B) DLBCL cells (left: WSU-DHL-2; right: U2932) were treated with CTX (20 μM), Spautin-1(10 μM), Combination or erastin (2 μM), and the GSH level was quantified by Micro GSH Assay Kit. (C) The xenografted tumors were removed from mice, cut into pieces and homogenized. The GSH level of the tumor tissue homogenate was determined by Micro GSH Assay Kit.


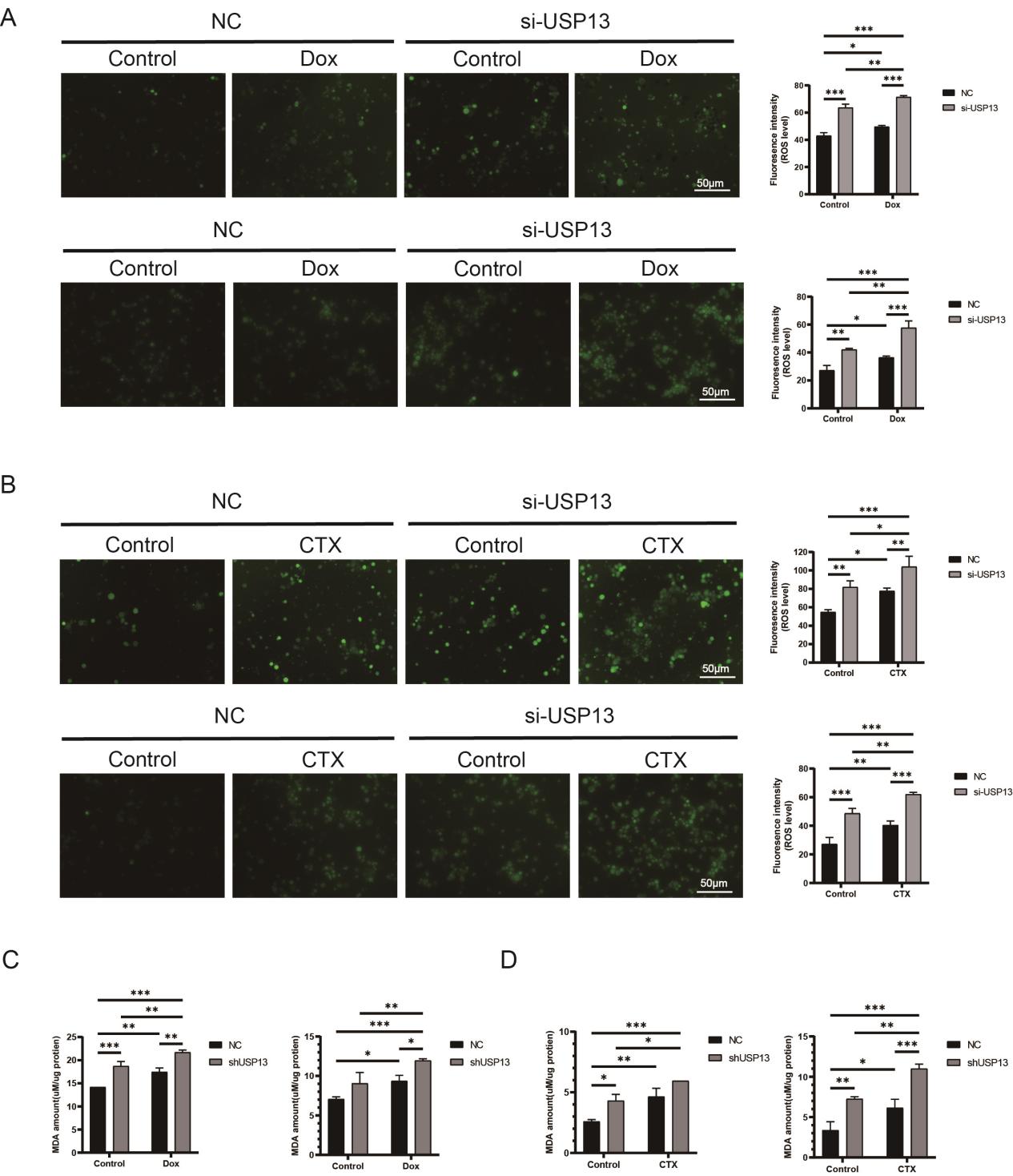


**Supplemental figure 7. Effect of USP13 knockdown on Dox/CTX-induced ROS and MDA increase in DLBCL cells.** (A) After knockdown of USP13 in DLBCL cells, the ROS level was quantified by inverted fluorescence microscope after treatment with Dox (1 μM) using DCFH-DA probe in SU-DHL-2 (upper) and WSU-DLCL-2 (lower) cells. The fluorescence intensity was visualized as bar chart (upper: SU-DHL-2; lower: WSU-DLCL-2). (B) After knockdown of USP13 in DLBCL cells, the ROS level was quantified by inverted fluorescence microscope after treatment with CTX (20 μM) using DCFH-DA probe in SU-DHL-2 (upper) and WSU-DLCL-2 cells (lower). The fluorescence intensity was visualized as bar chart (upper: SU-DHL-2; lower: WSU-DLCL-2). (C) After knockdown of USP13 in DLBCL cells, the lipid peroxide level was examined after treatment with Dox (1 μM) for 48 h using Lipid Peroxidation MDA Assay Kit in U2932 (left) or TMD8 (right) cells. (D) After knockdown of USP13 in DLBCL cells, the lipid peroxide level was examined after treatment with CTX (20 μM) for 48 h using Lipid Peroxidation MDA Assay Kit in U2932 (left) or TMD8 (right) cells.

**Supplemental figure 8**. **H&E staining of heart, liver, spleen, lung and kidney tissues after Spautin-1/Dox/CTX treatment.** Heart: The structure of myocardium, endocardial membrane and epicardium was clear in control group and administration group, and no obvious abnormality was found in cardiomyocytes and interstitium. Sp+Dox, Sp+CTX group occasionally observed myocardial cell vacuoles (orange arrow). Liver: A small amount of hepatocyte edema (blue arrow) was observed in all 4 groups, and no obvious inflammatory cell infiltration was observed. Hepatocyte necrosis was occasionally seen in the control and Spautin-1 groups (black arrow). Vascular congestion was occasionally observed in control and Sp+Dox groups. (Red arrow). Hepatic sinus congestion and dilatation were occasionally observed in Sp+Dox group (orange arrow). Spleen: There were more granulocytes and a few multinucleated cells scattered in the spleen tissues of the four groups (blue arrow). A small amount of granulocyte infiltration is seen in the control and the Spautin-1 groups (red arrow). Lung: The lung tissue of the four groups had a small amount of congestion, and the bronchiole epithelial cell structure was clear. The control and the Spautin-1 groups had small bleeding (red arrow). Eosinophilic substances were occasionally found in lumen of Sp+CTX group (blue arrow). Kidney: In the kidney tissues of the four groups, the glomeruli were evenly distributed, the number of cells in the glomeruli was uniform and the matrix was uniform. There was a small amount of blood stasis between the urinary tubules (orange arrow) and no obvious inflammatory cell infiltration was observed. Watery degeneration of renal tubular epithelial cells was occasionally observed in Spautin-1 and Sp+Dox groups (blue arrow).
