# Supplementary material for: USP13 dictates Ran turnover and vulnerability to ferroptosis in diffuse large B cell lymphoma (DLBCL)
Source: Cell Death Dis. 2025 Nov 28;16(1):870. doi: 10.1038/s41419-025-08207-6 (PMC12663190; doi:10.1038/s41419-025-08207-6)
Supplement: Supplementary file 2 — WB original data [file 41419_2025_8207_MOESM2_ESM.pdf]

Original blot

USP13 dictates Ran turnover and vulnerability to ferroptosis in diffused large B cell lymphoma (DLBCL)

Figure 1G

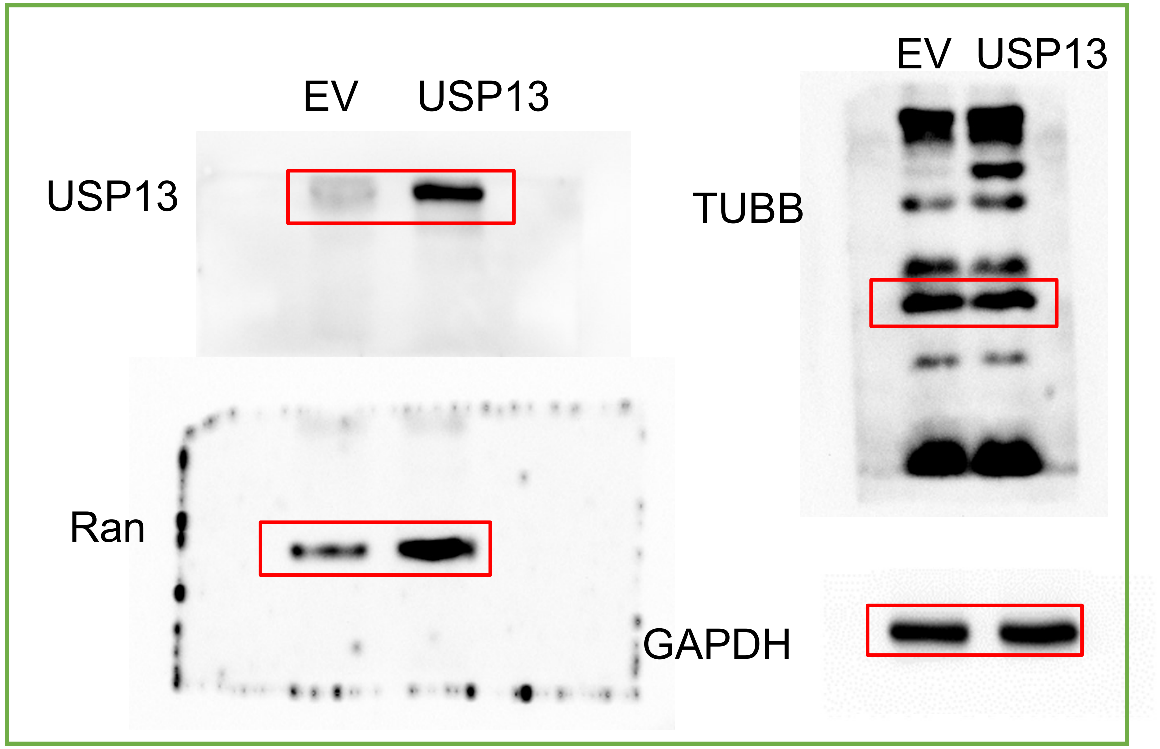

Figure 1K

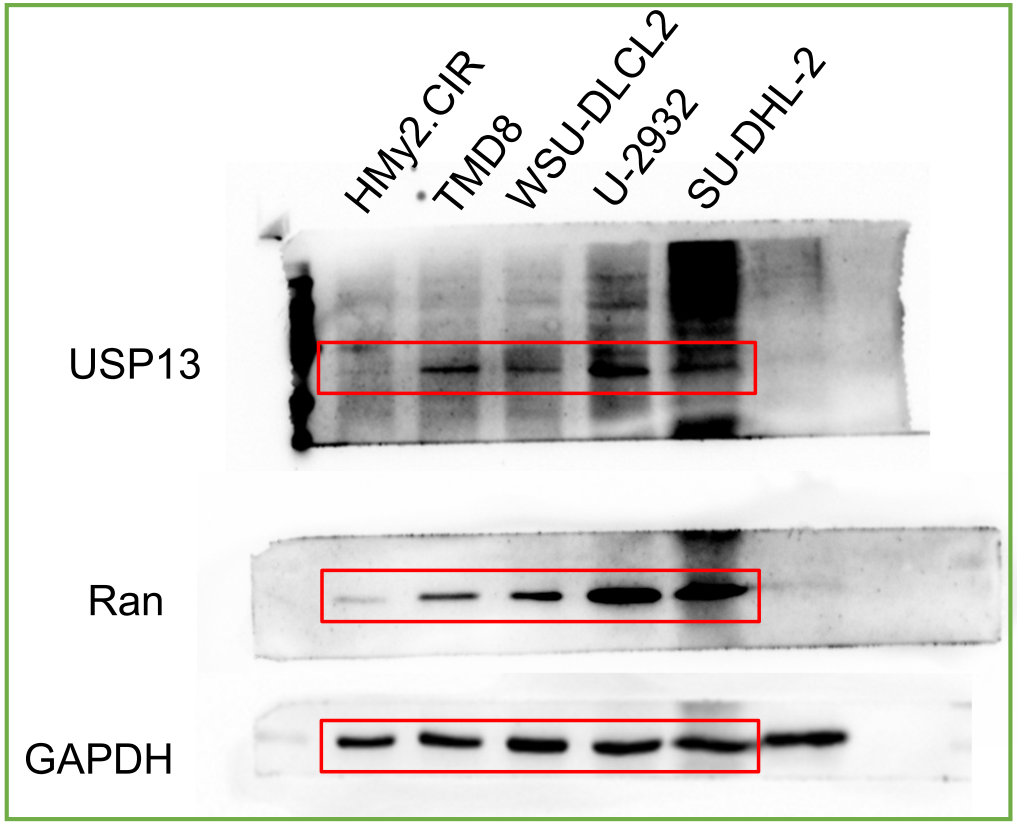

Figure 2A

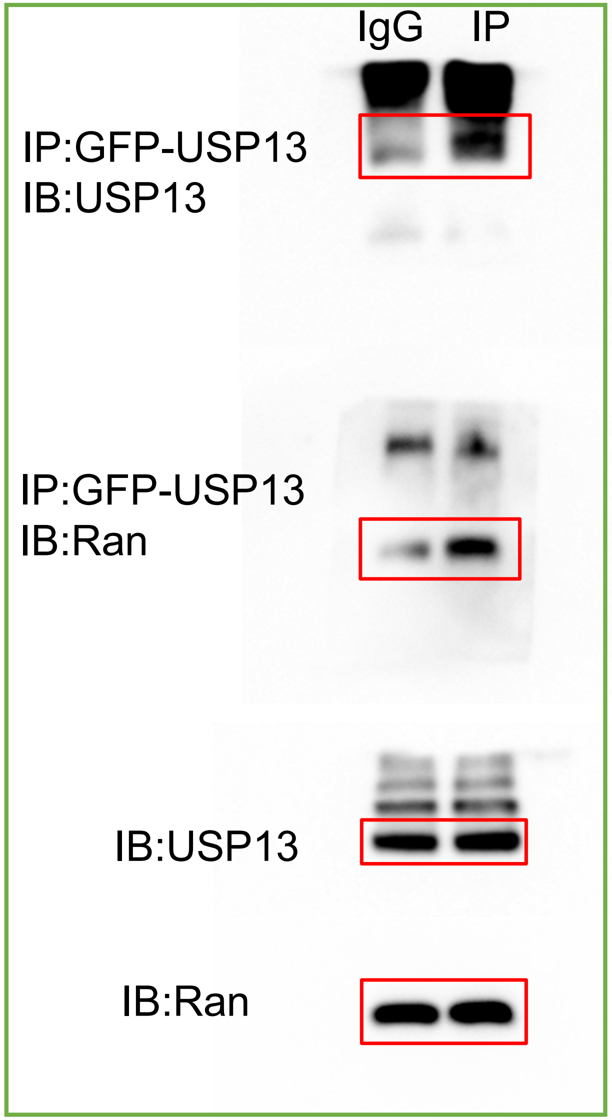

Figure 2B

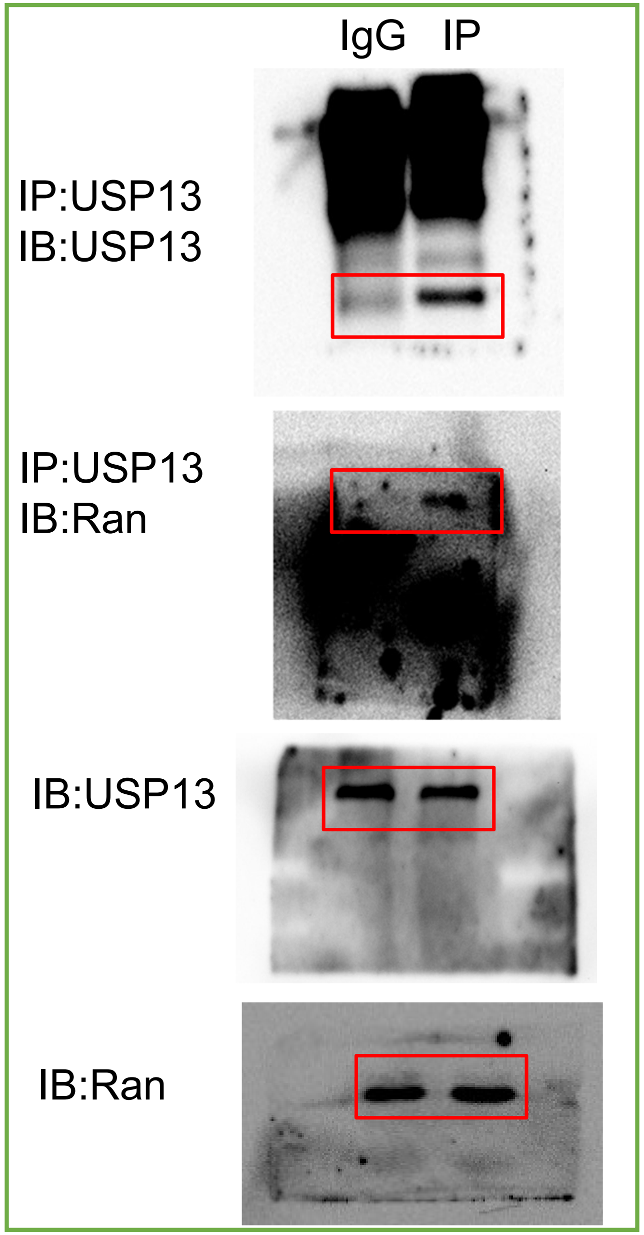

Figure 2C

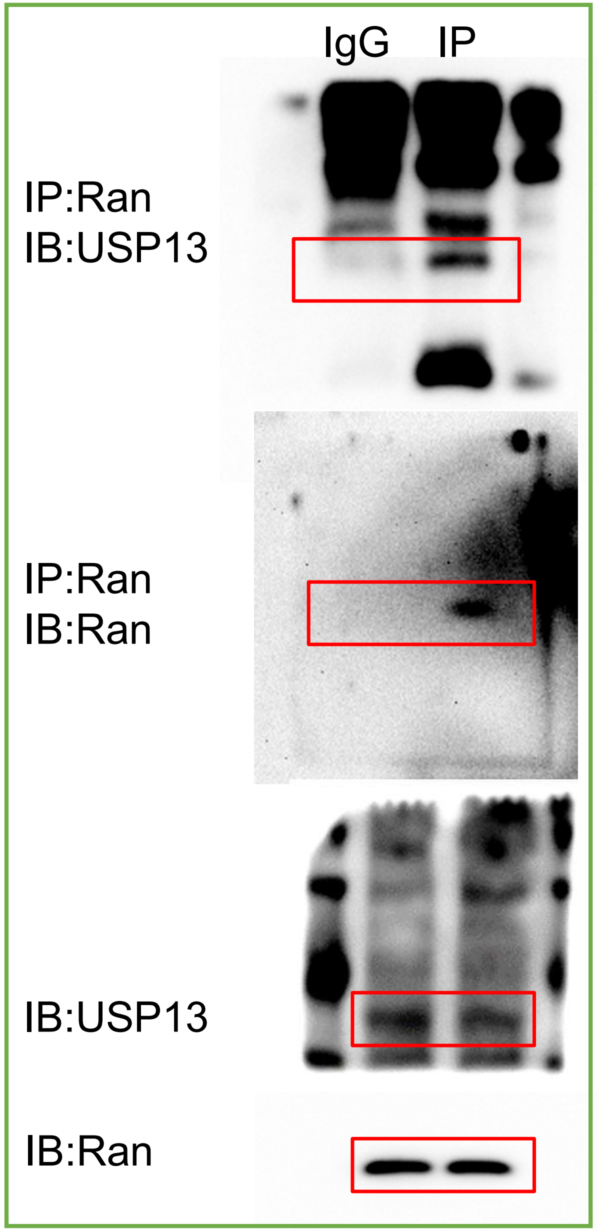

Figure 2D

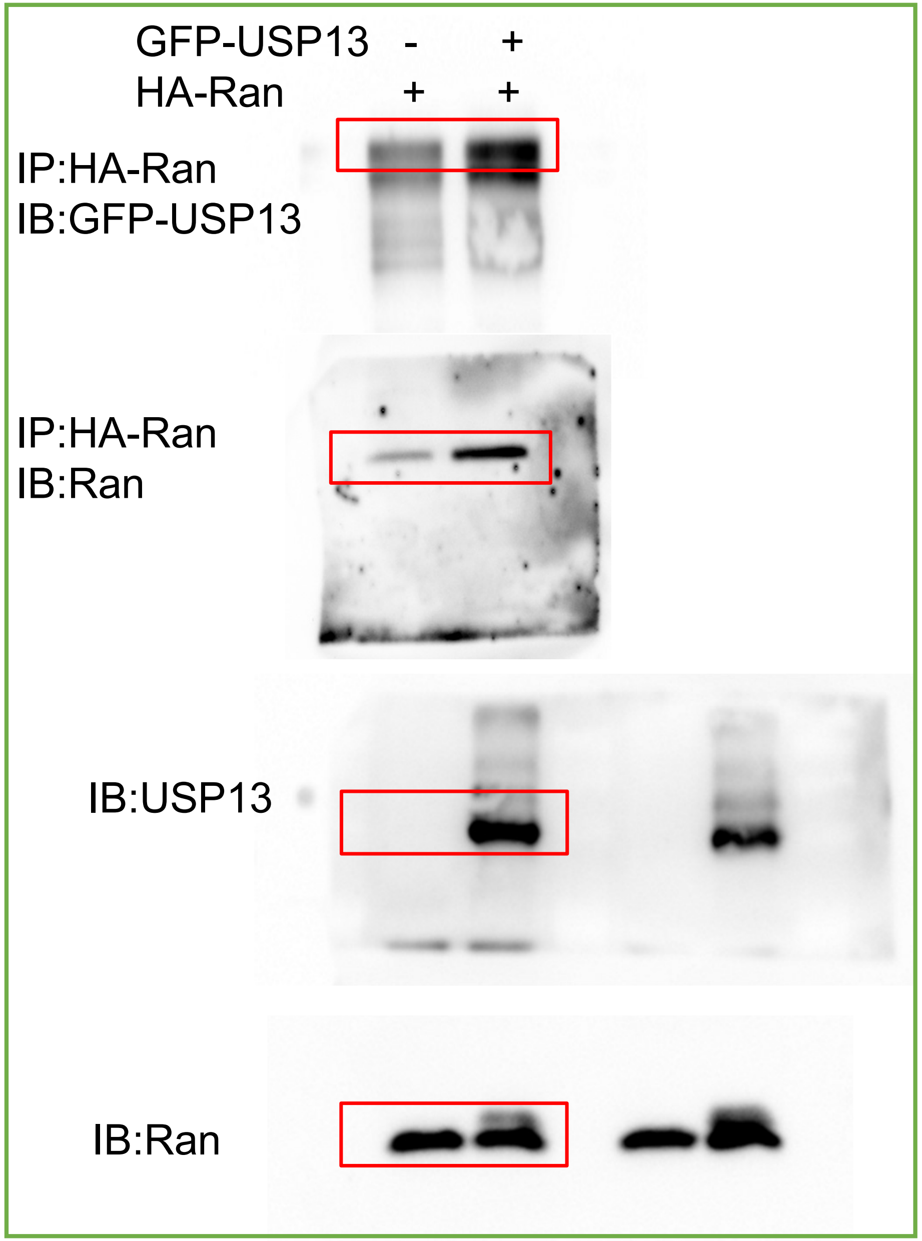

Figure 2E

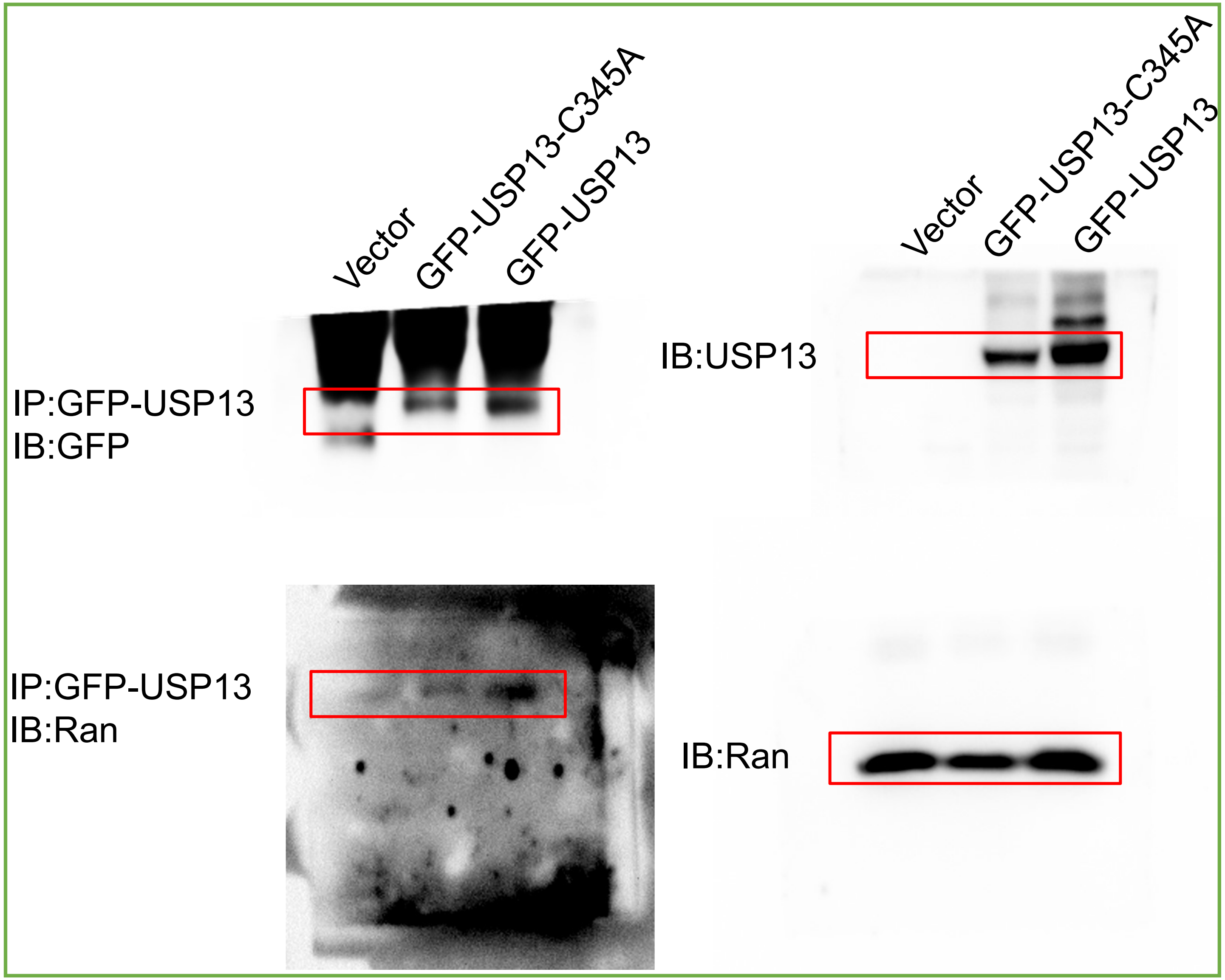
Vector
GFP-USP13-C345A
GFP-USP13
IP:GFP-USP13
IB:GFP
Vector
GFP-USP13-C345A
GFP-USP13
IB:USP13
IP:GFP-USP13
IB:Ran
IB:Ran

Figure 2G

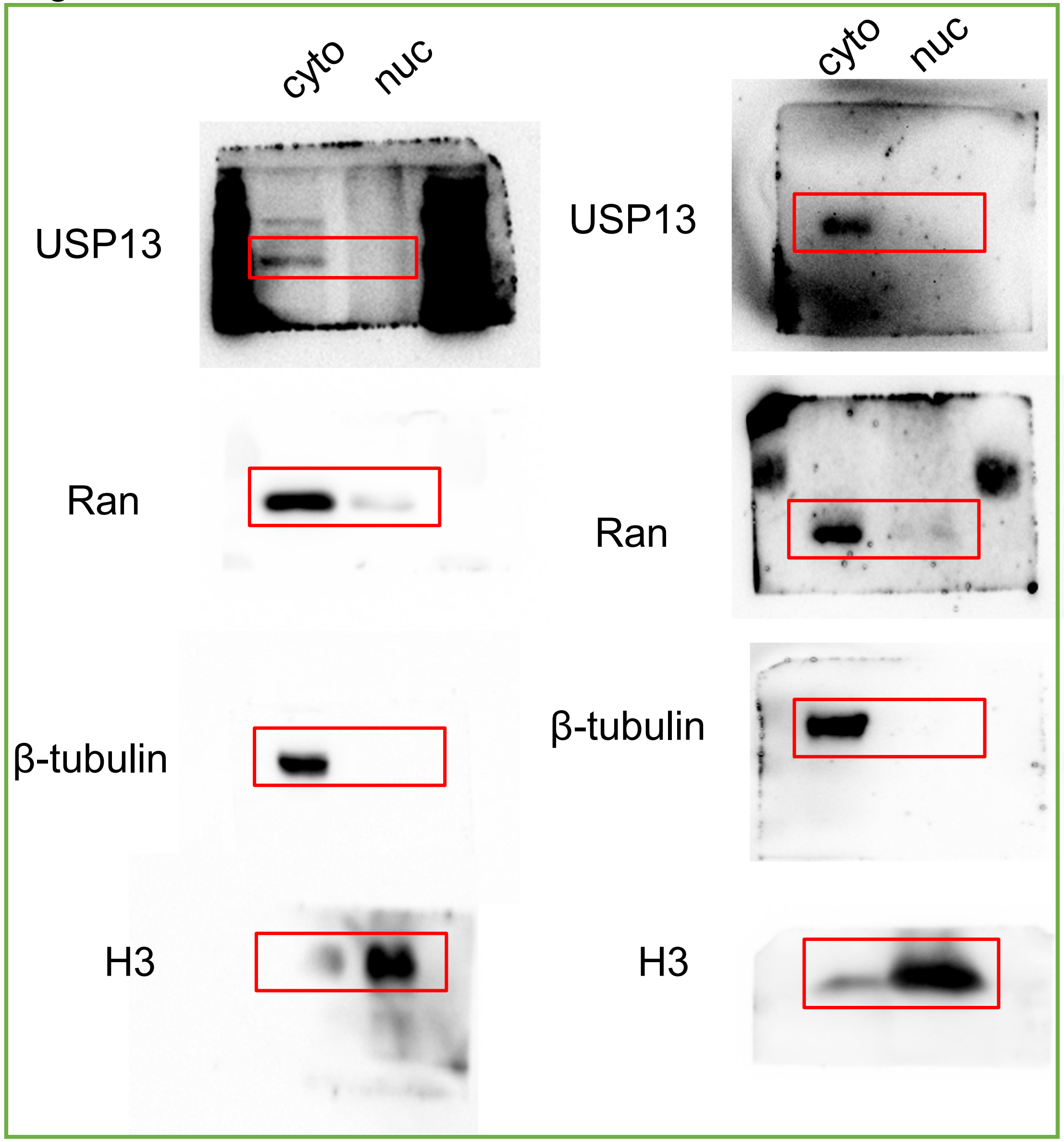
cyto
nuc
USP13
Ran
β-tubulin
H3
cyto
nuc
USP13
Ran
β-tubulin
H3

Figure 2H

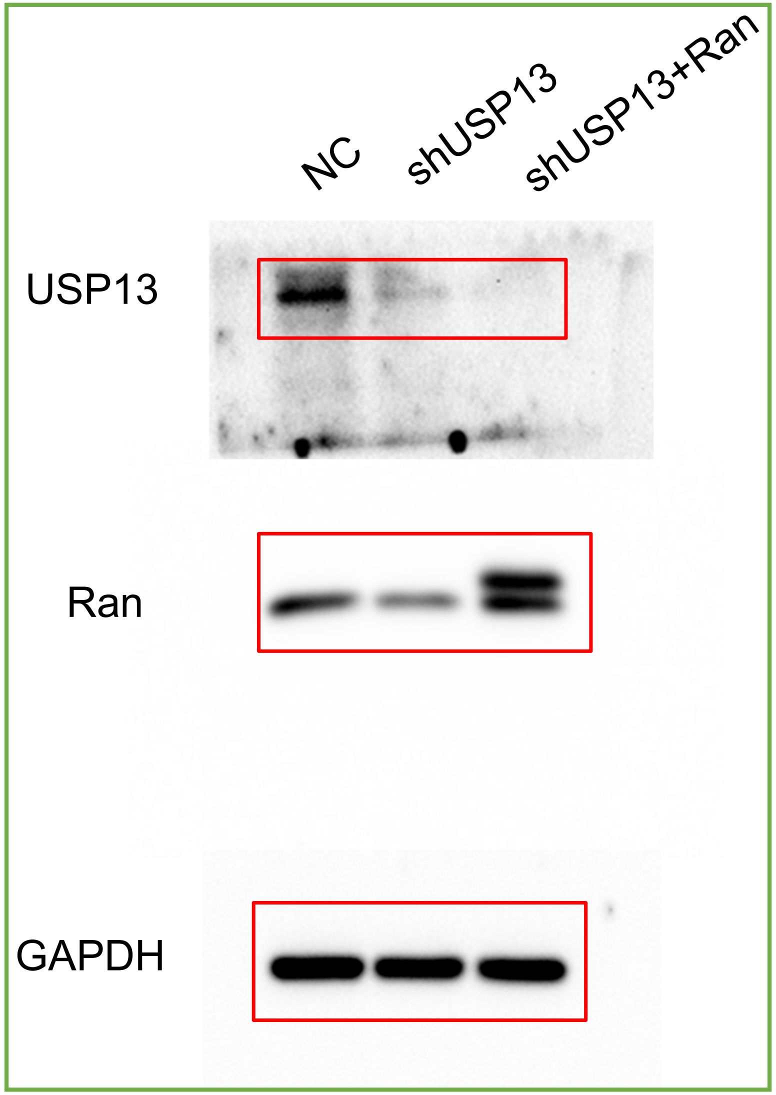

Figure 2I

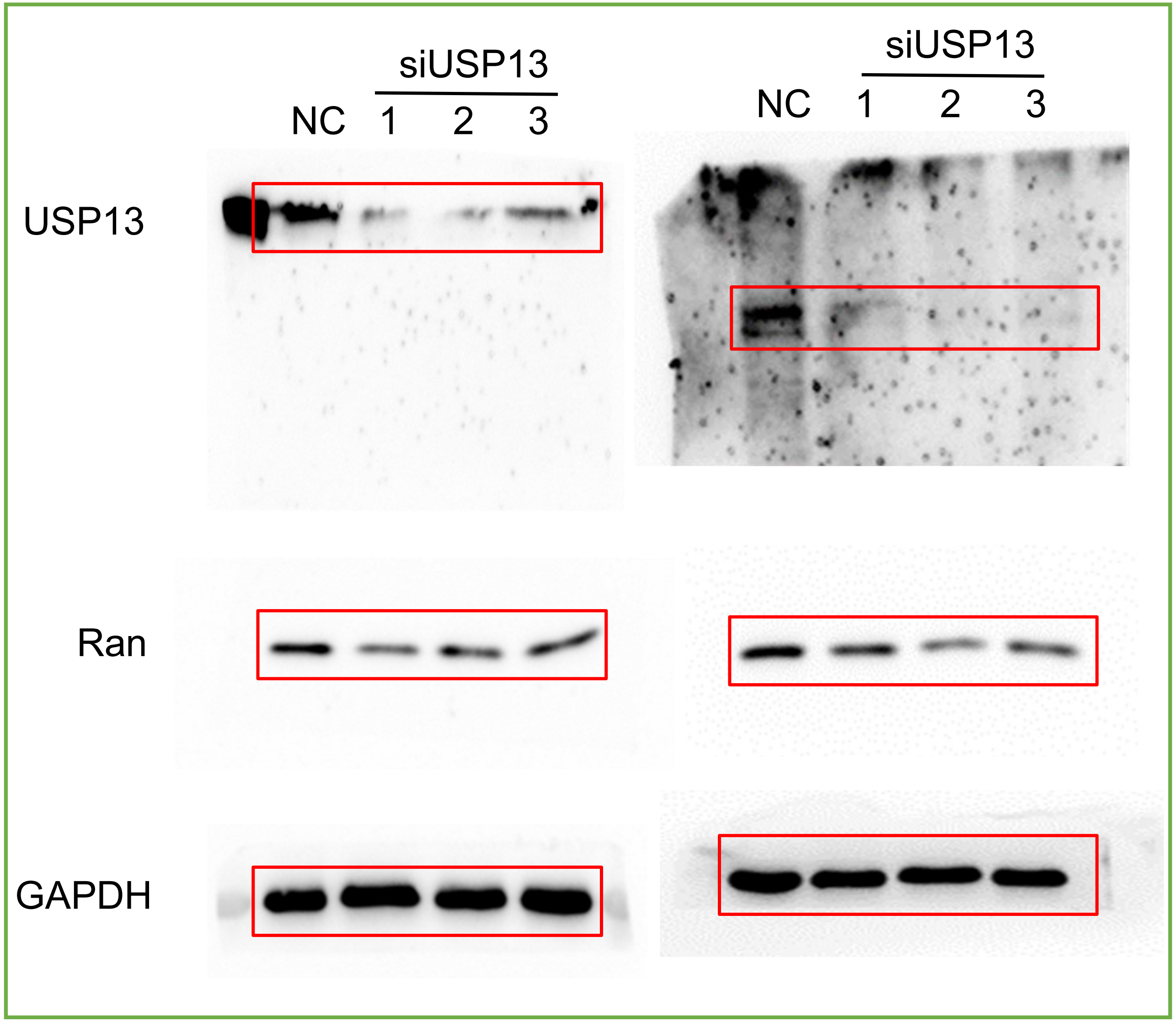

Figure 2K

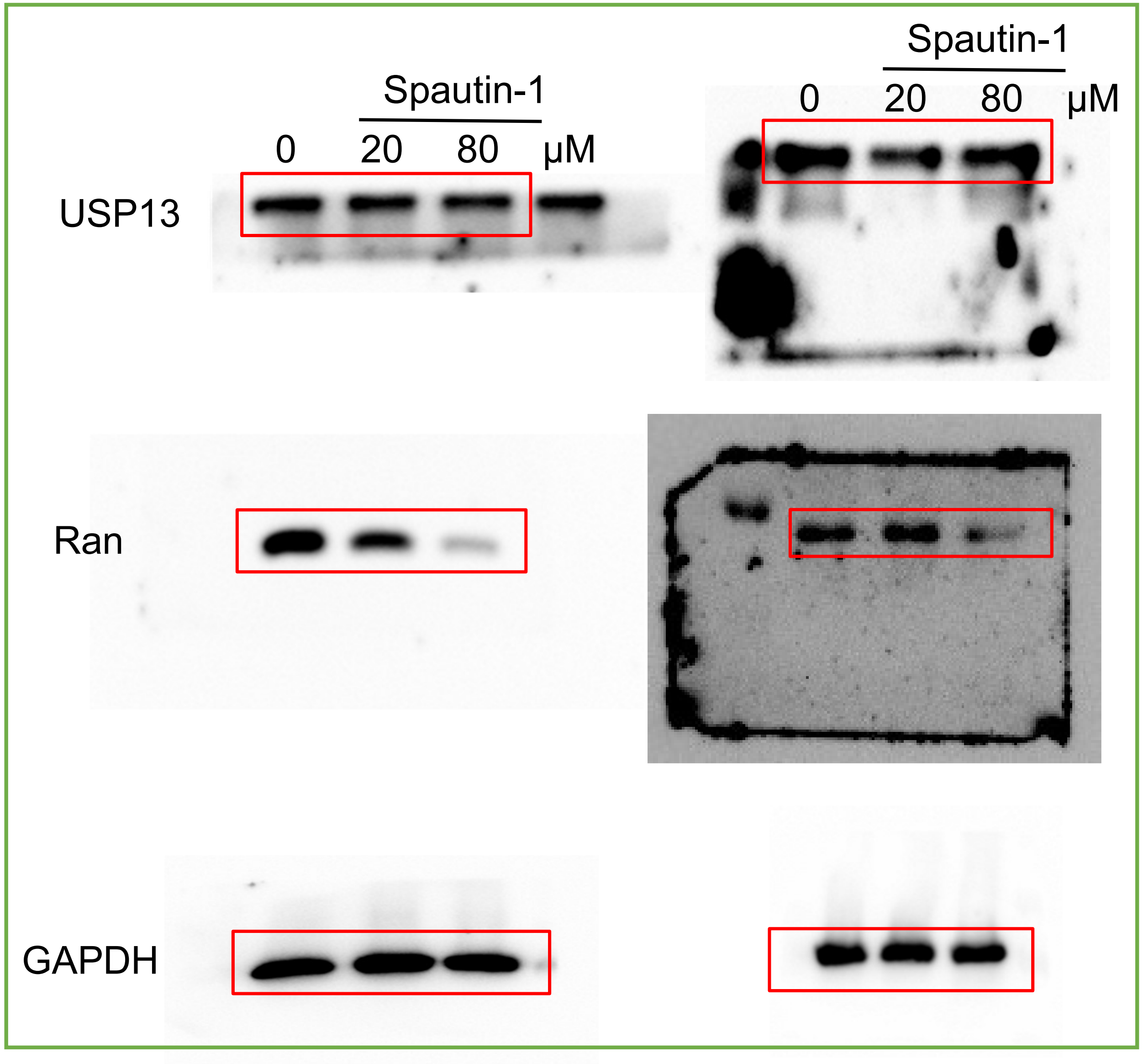

Figure 2L

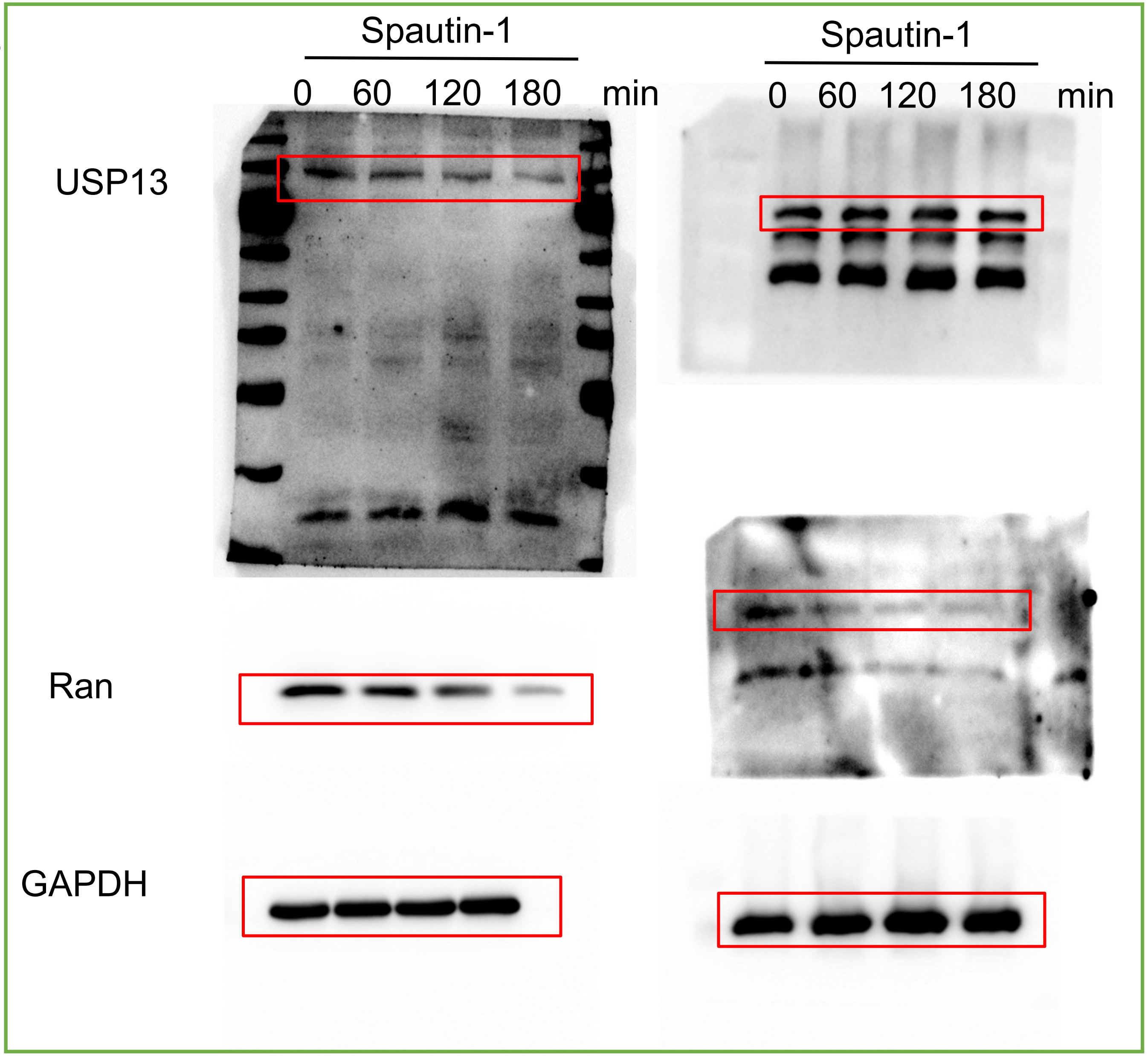
Spautin-1
0 60 120 180 min
Spautin-1
0 60 120 180 min
USP13
Ran
GAPDH

Figure 2M

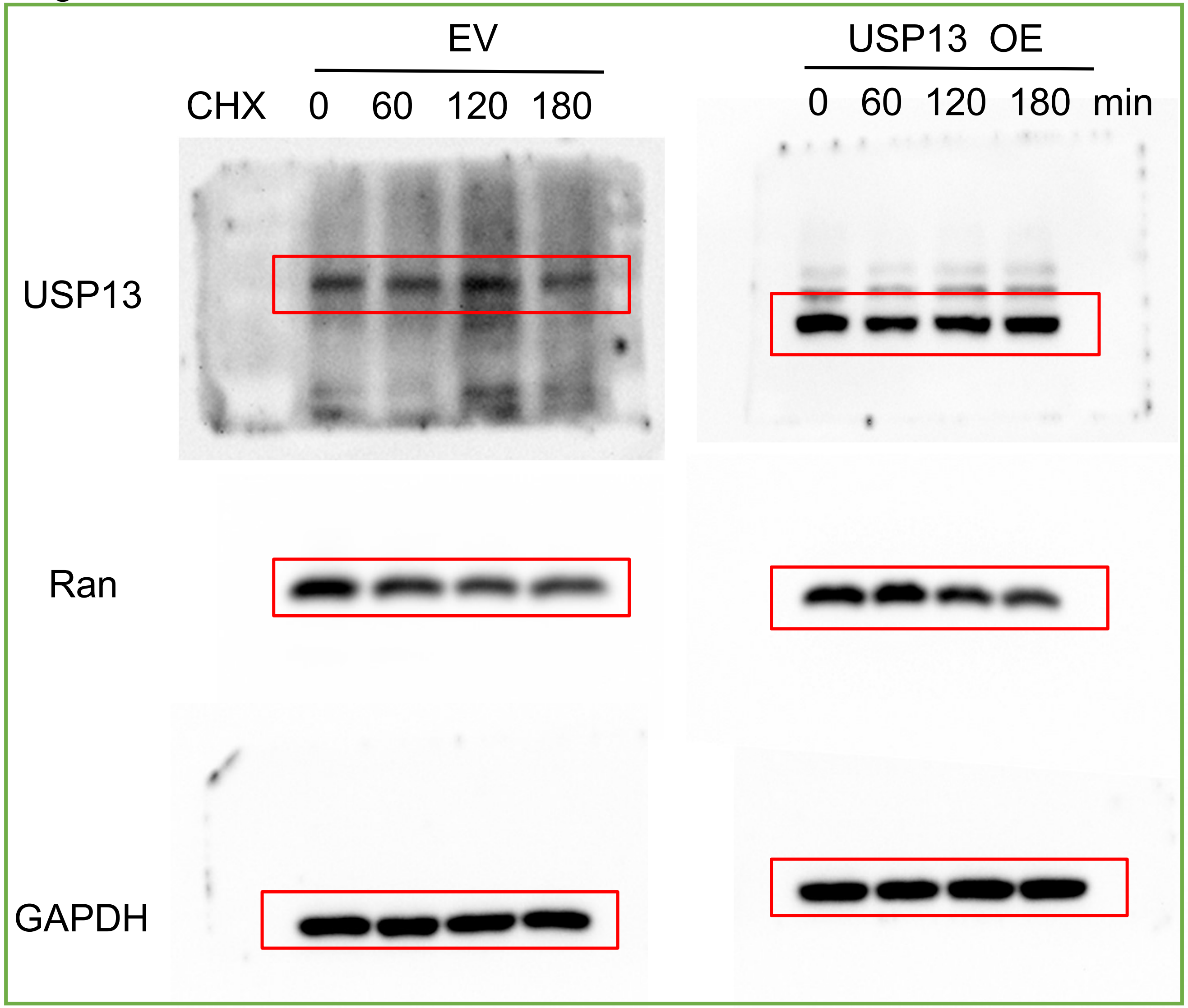

Figure 2N

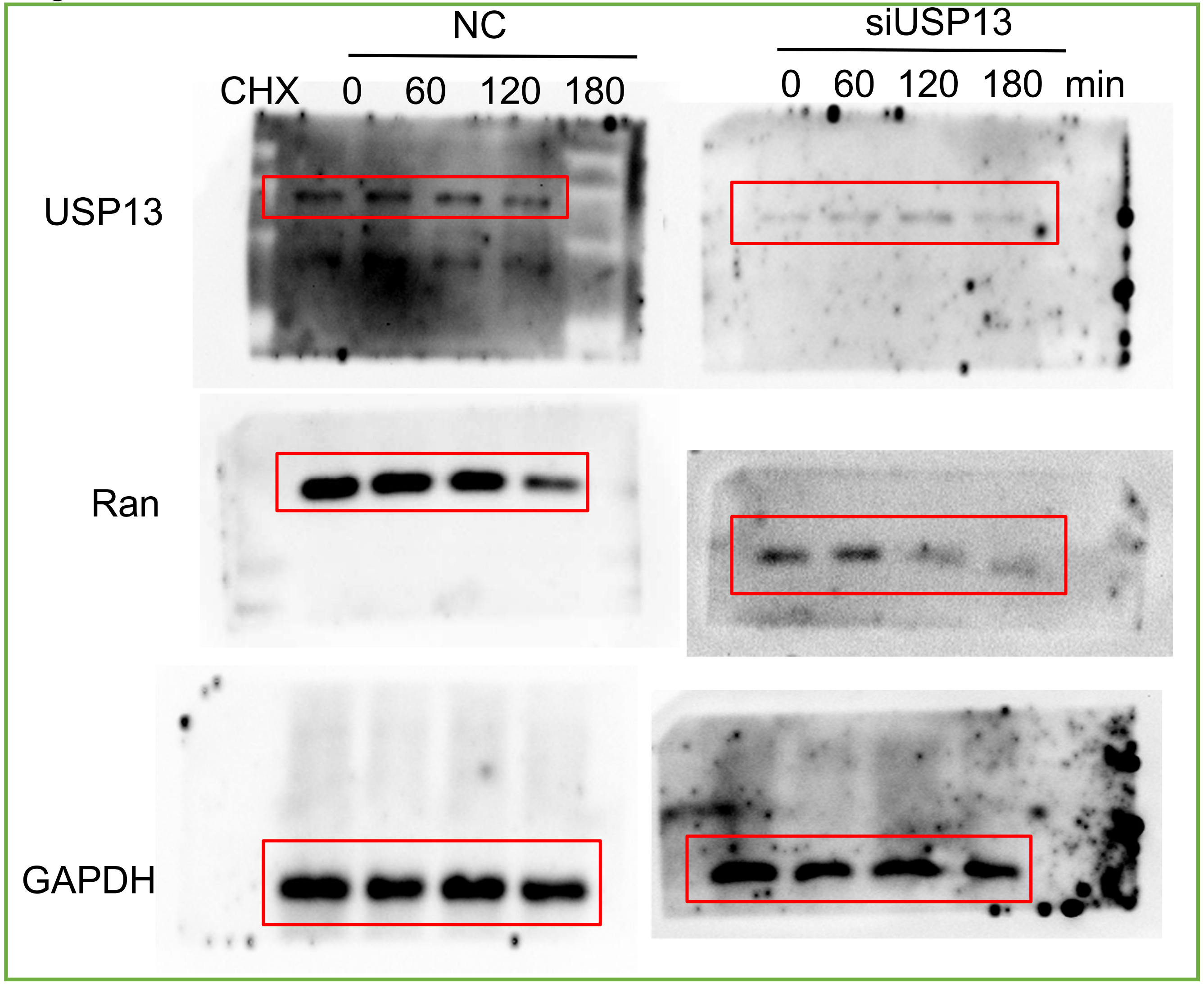

Figure 2O

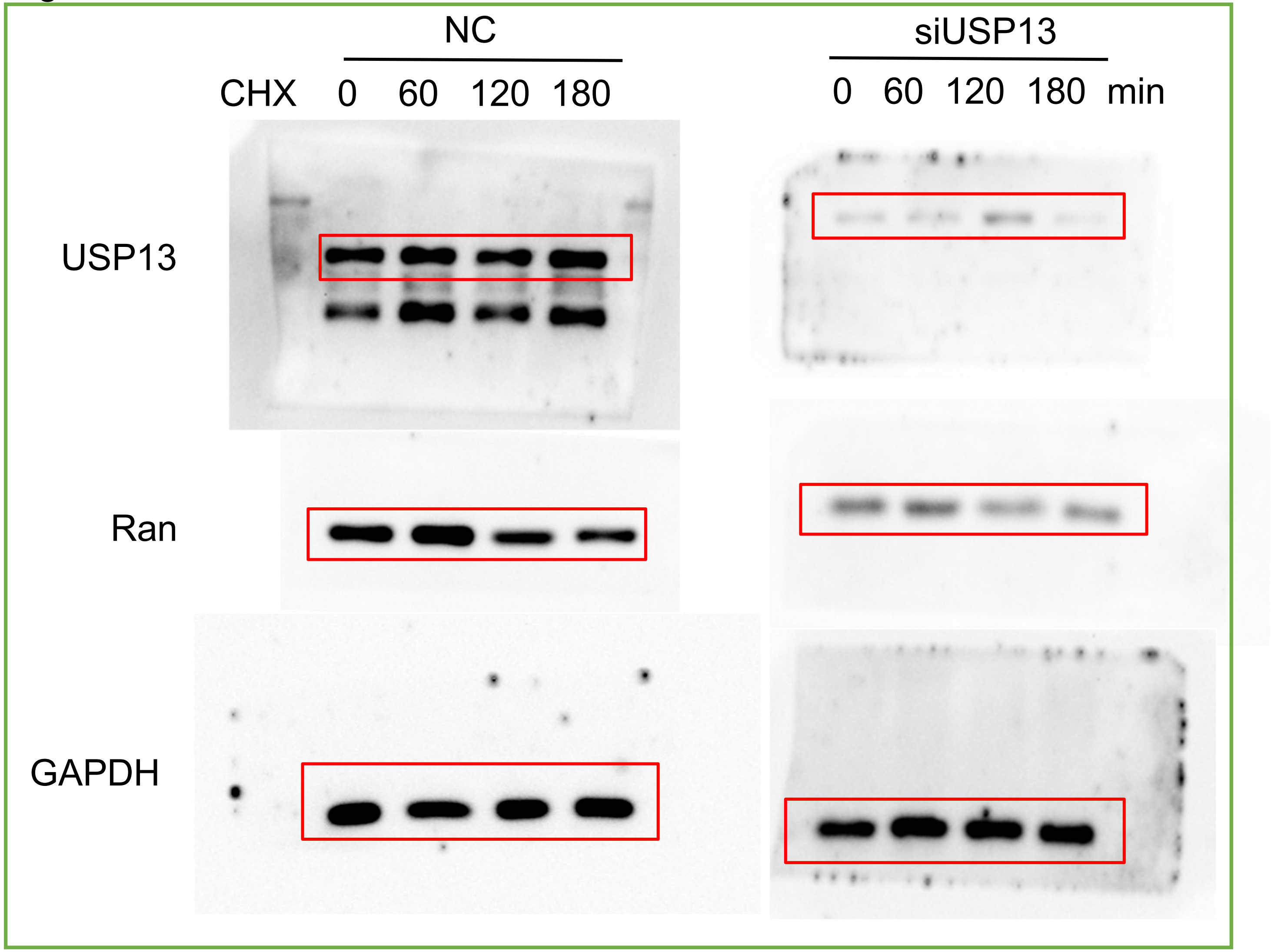

Figure 3A

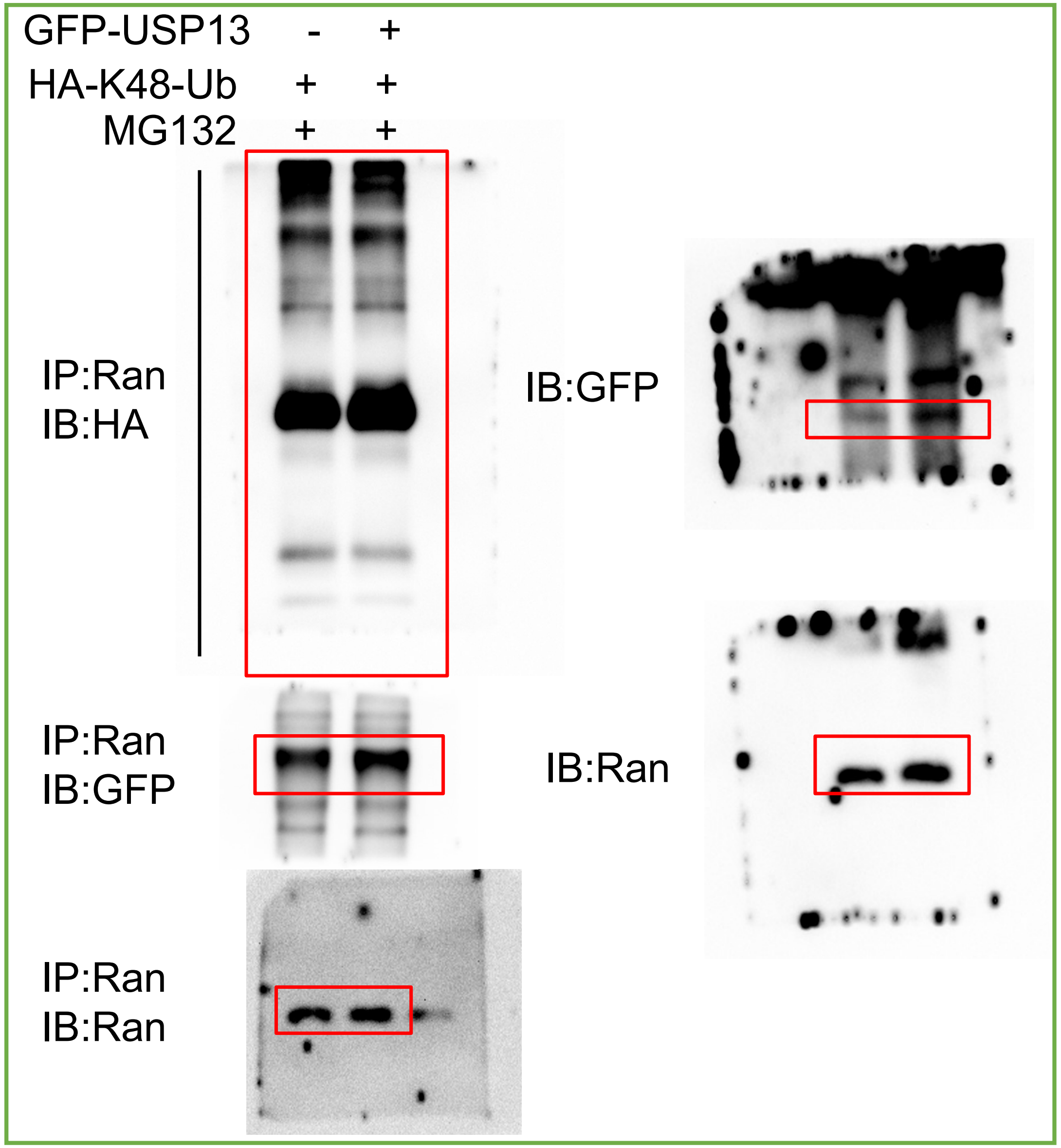

GFP-USP13 - +
HA-K48-Ub + +
MG132 + +
IP:Ran
IB:HA
IB:GFP
IP:Ran
IB:GFP
IB:Ran
IP:Ran
IB:Ran

Figure 3B

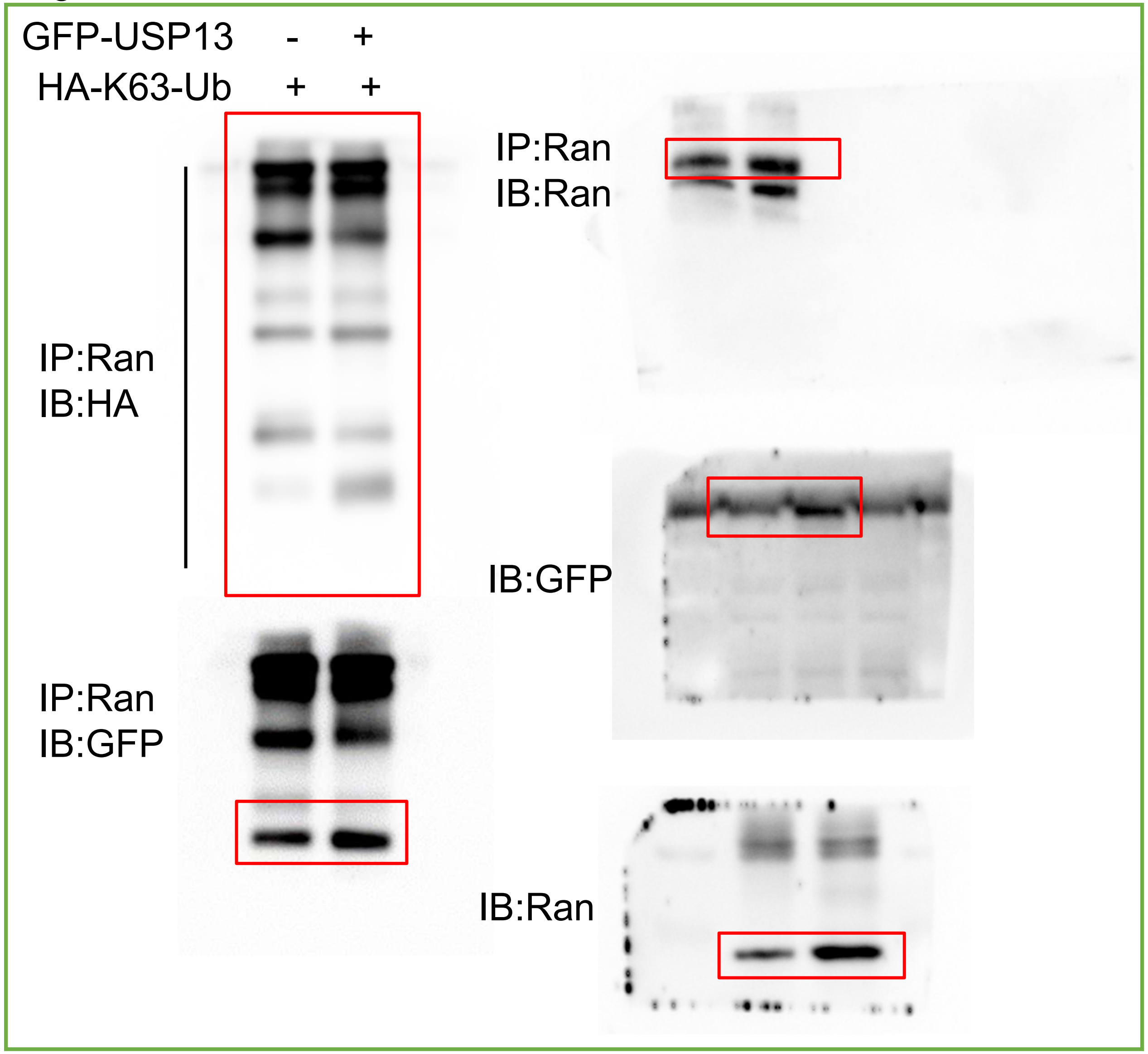

GFP-USP13 - +
HA-K63-Ub + +
IP:Ran
IB:HA
IP:Ran
IB:GFP
IP:Ran
IB:Ran
IB:GFP
IB:Ran

Figure 3C

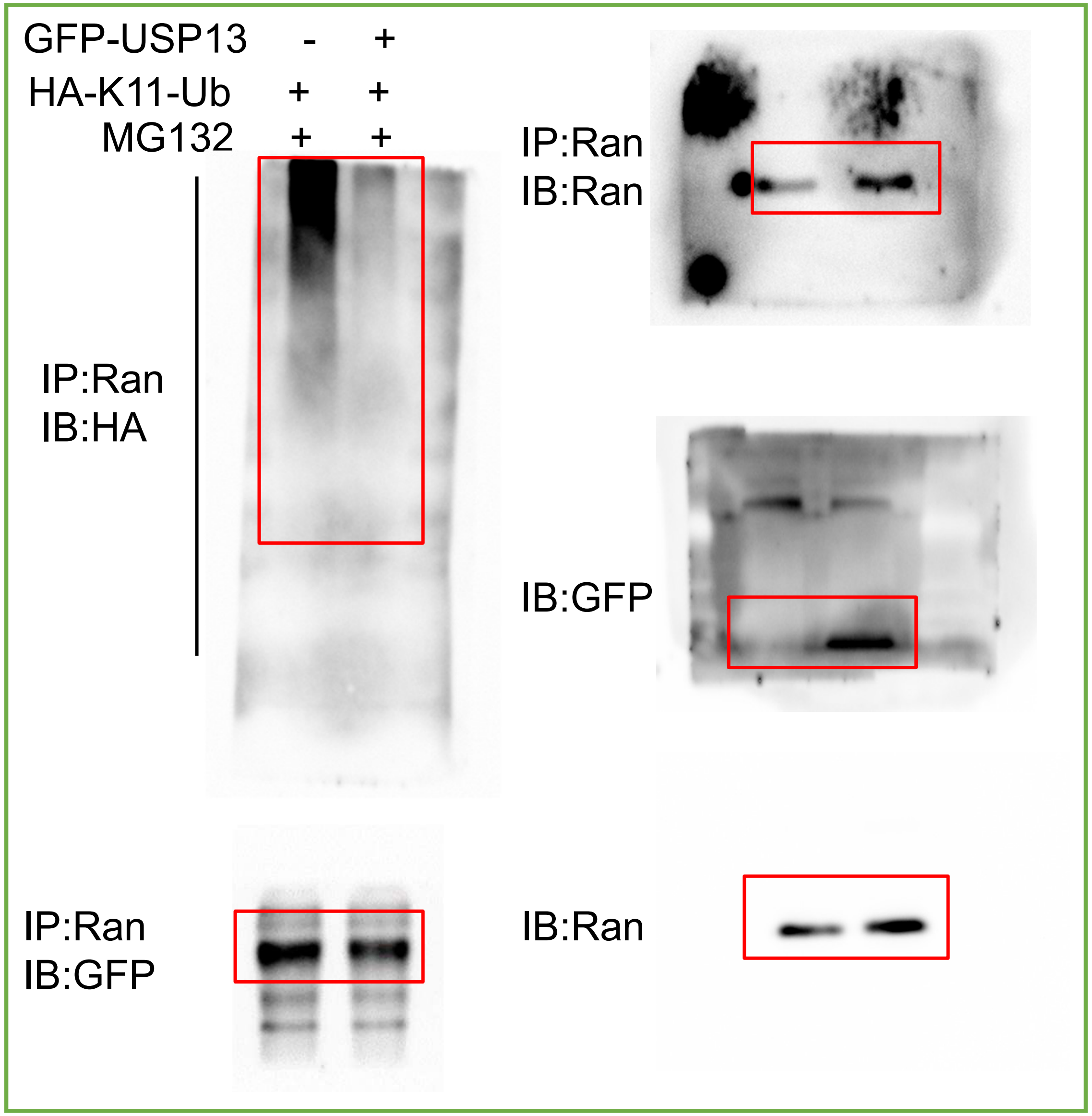

GFP-USP13 - +
HA-K11-Ub + +
MG132 + +
IP:Ran
IB:HA
IP:Ran
IB:GFP
IP:Ran
IB:Ran
IB:GFP
IB:Ran

Figure 3D

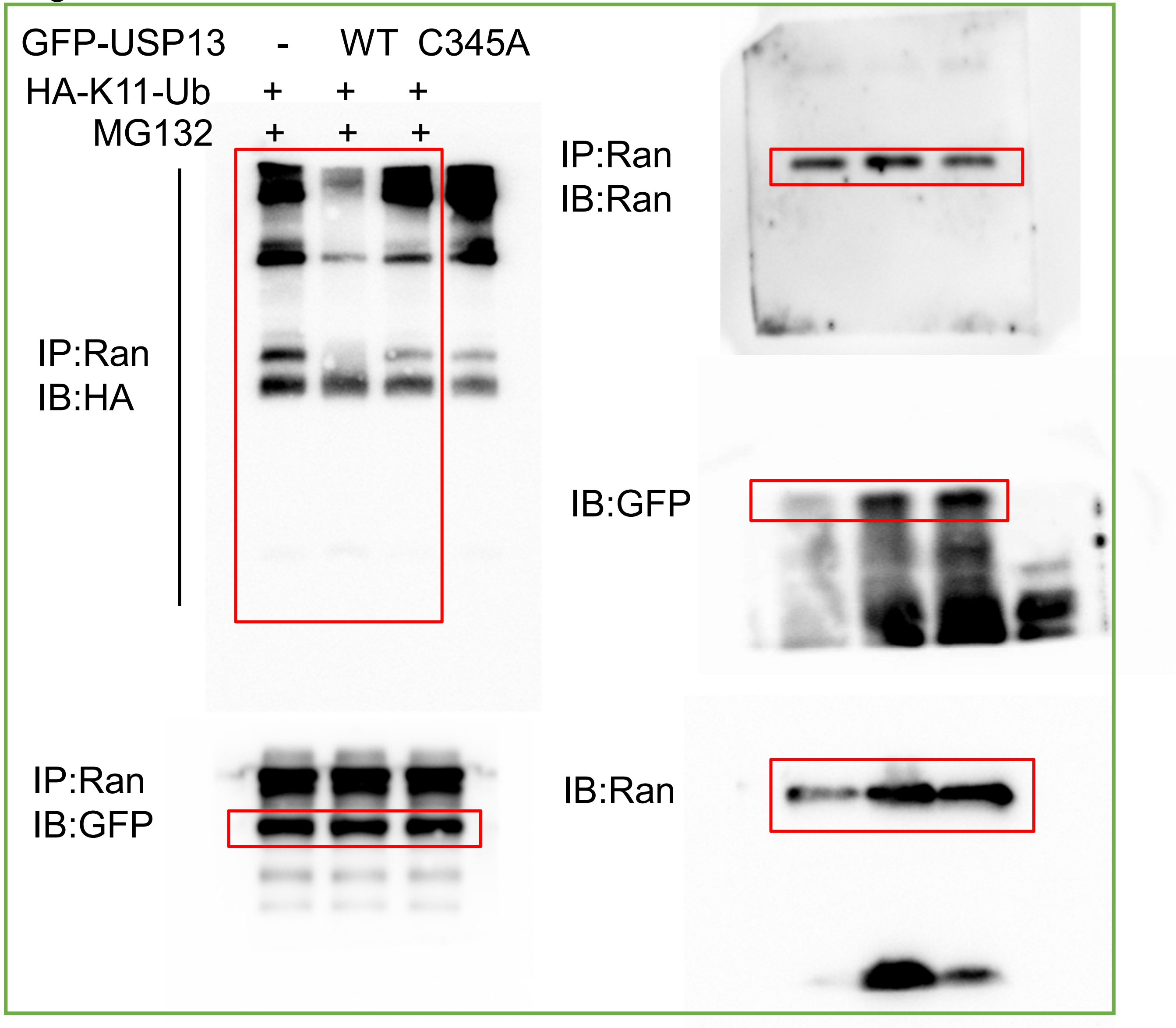
GFP-USP13 - WT C345A
HA-K11-Ub + + +
MG132 + + +
IP:Ran
IB:HA
IP:Ran
IB:Ran
IB:GFP
IP:Ran
IB:GFP
IB:Ran

Figure 3E

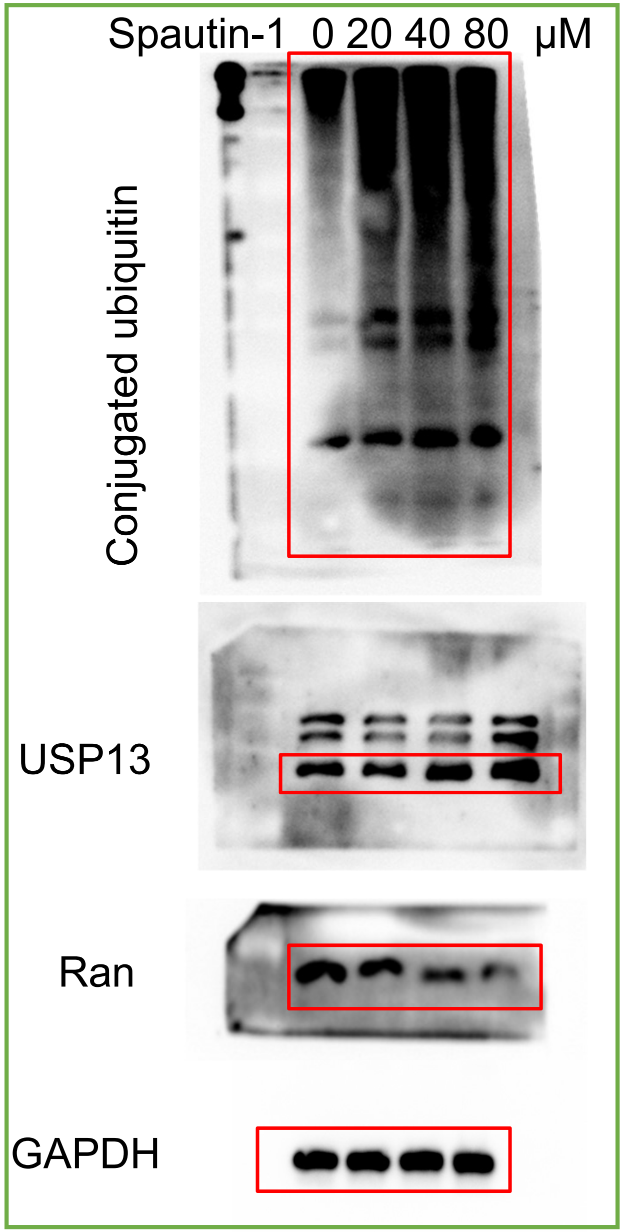

Figure 3F

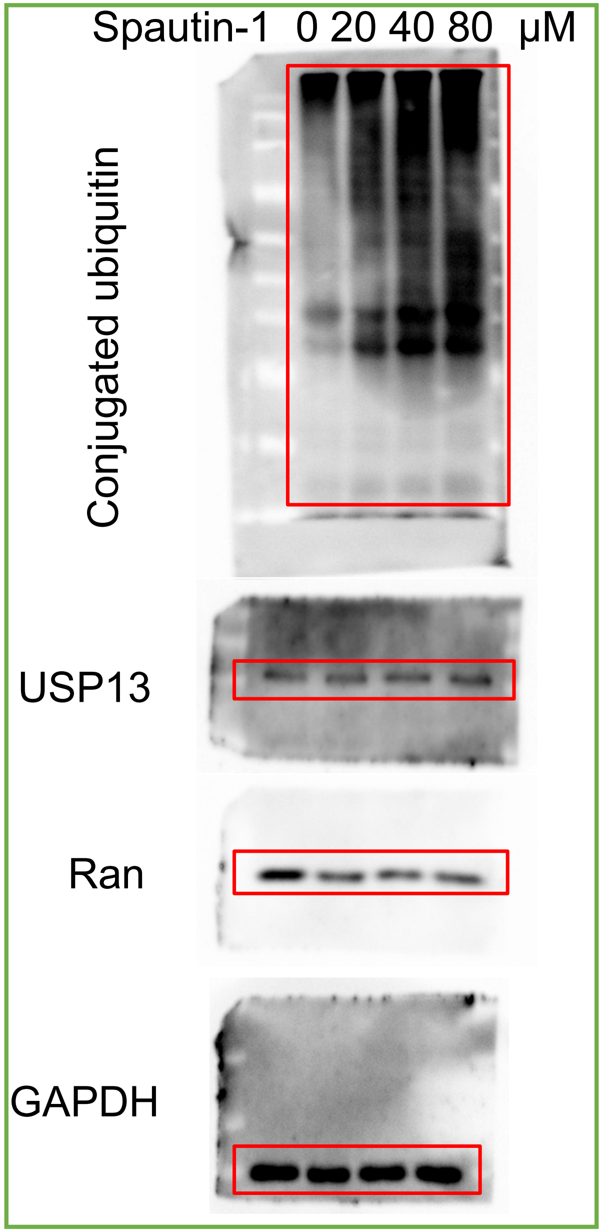

Figure 3G

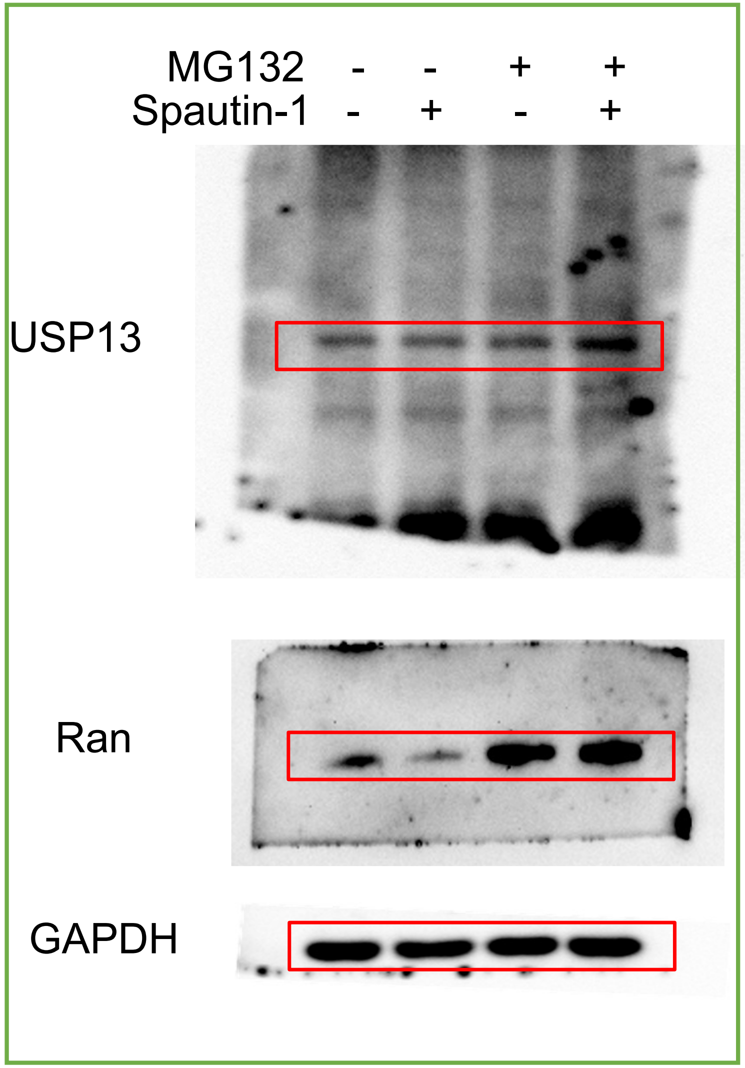

Figure 3H

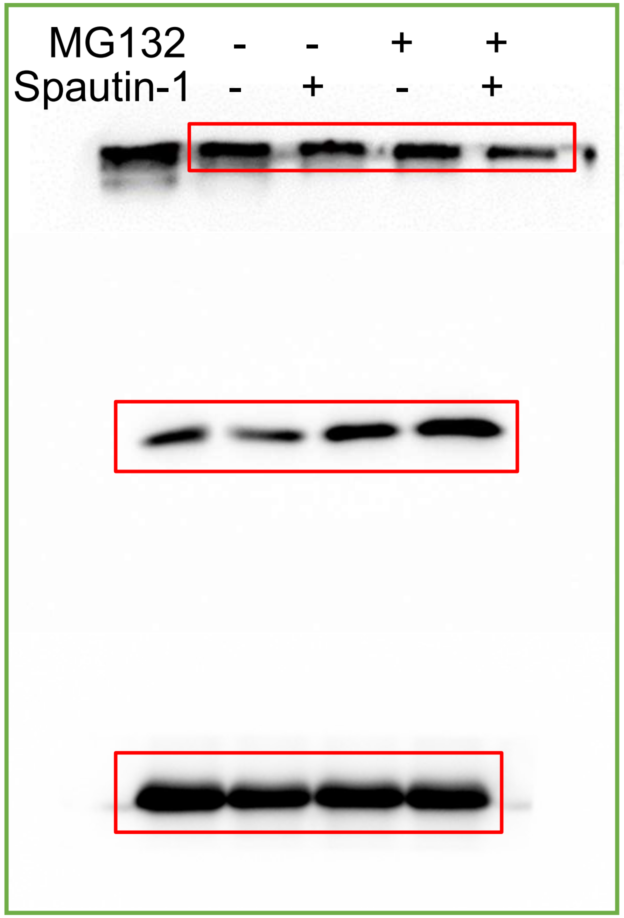

Supplemental figure 2

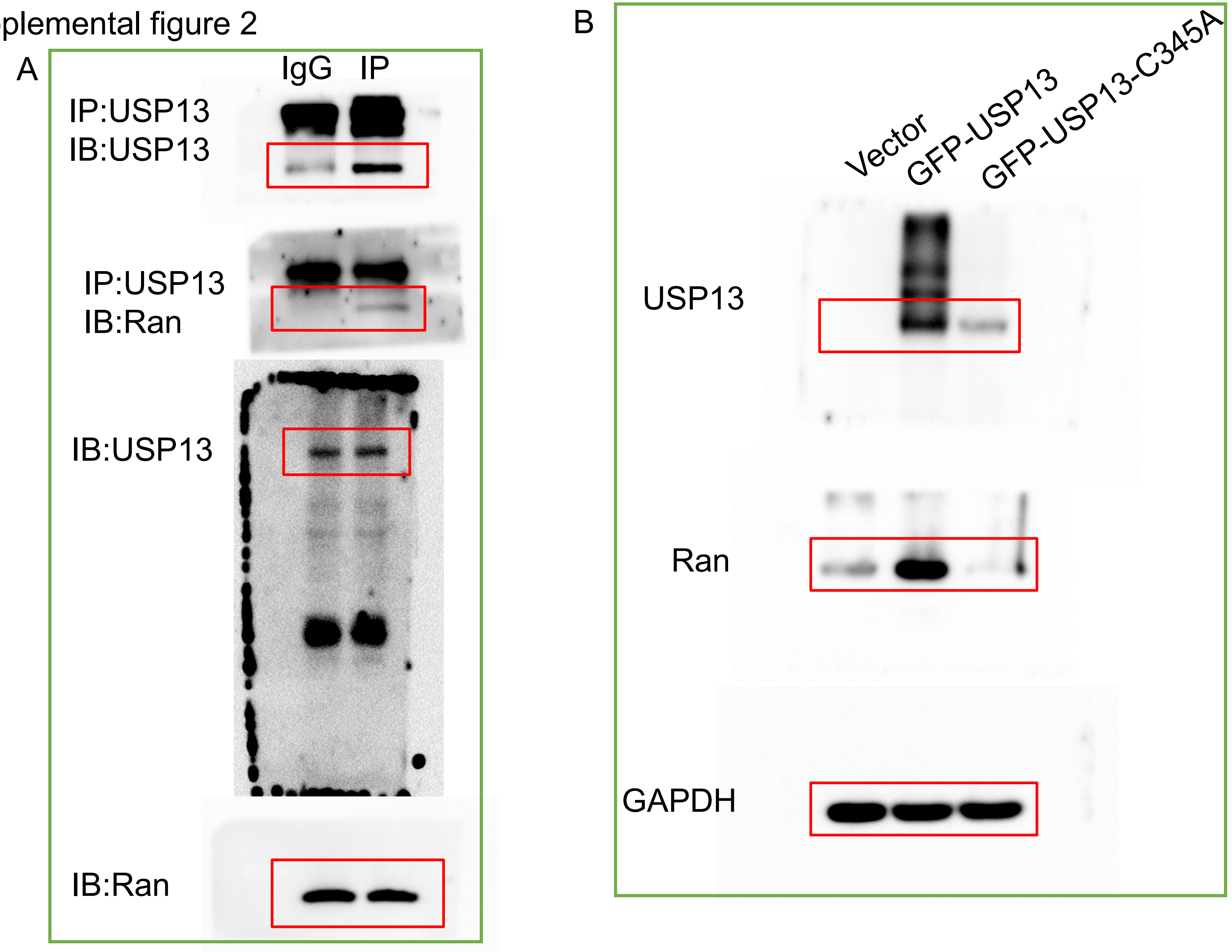

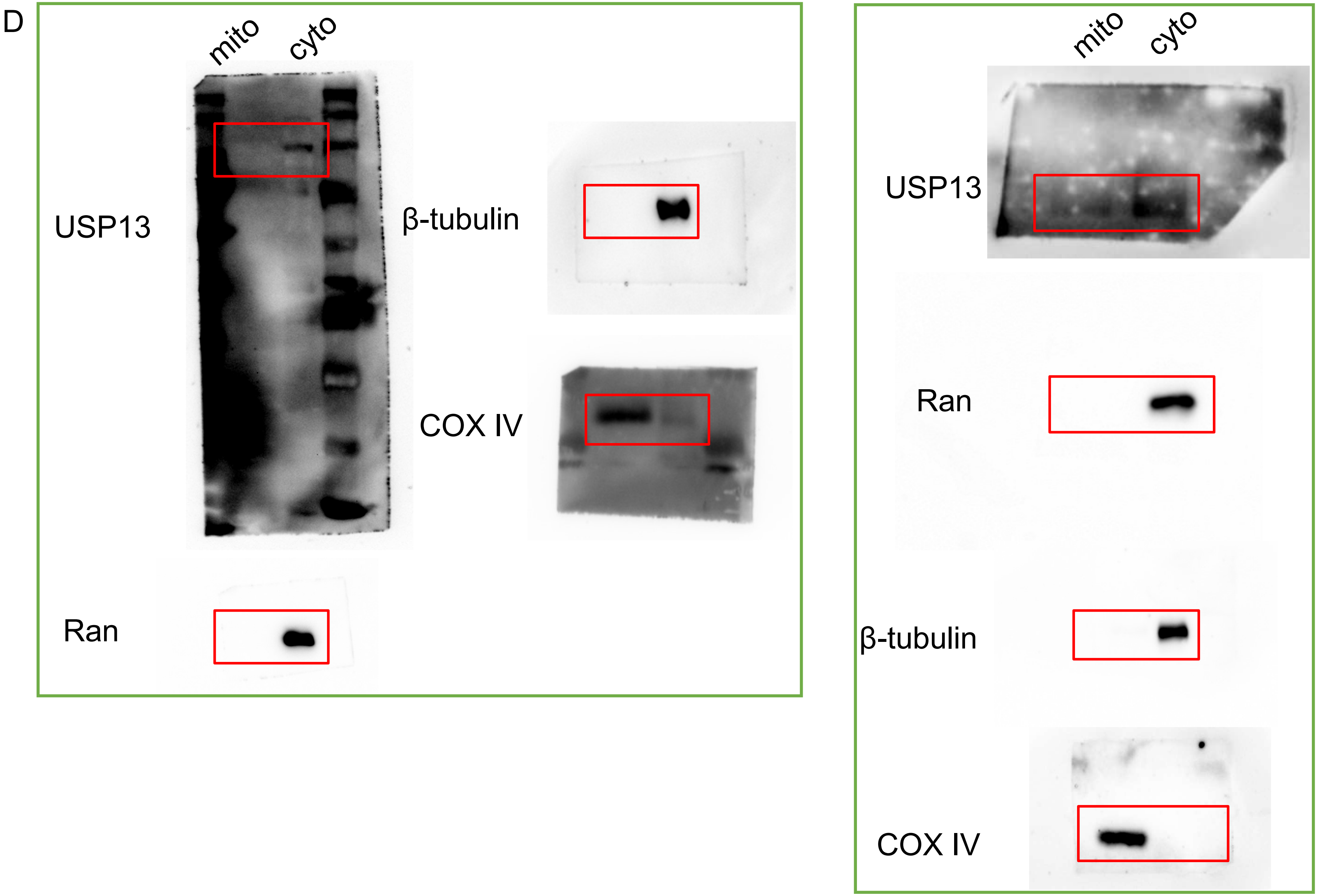
D
mito
cyto
USP13
β-tubulin
COX IV
Ran
mito
cyto
USP13
Ran
β-tubulin
COX IV
